# The Neuro-Cardiac Symbiotic Engine: A Multimodal Fusion Architecture for Cognitive State Decoding via High-Performance Computing

**DOI:** 10.3390/life16050830

**Published:** 2026-05-18

**Authors:** Nayeli Bastidas-Benalcazar, Julián A. Calero-Apunte, Diego Almeida-Galarraga, Paulo Navas-Boada, Omar Alvarado-Cando, Andrés Tirado-Espín, Fernando Villalba-Meneses, Henry Carvajal Mora, Nathaly Orozco Garzón

**Affiliations:** 1 School of Biological Sciences and Engineering, Universidad Yachay Tech, San Miguel de Urcuquí 100119, Ecuador; nayeli.bastidas@yachaytech.edu.ec (N.B.-B.); julian.calero@yachaytech.edu.ec (J.A.C.-A.); dalmeida@yachaytech.edu.ec (D.A.-G.); pnavas@yachaytech.edu.ec (P.N.-B.); gvillalba@yachaytech.edu.ec (F.V.-M.); 2 Psy-Brain Group, Facultad de Informática y Ciencias de la Computación, Universidad Catolica de Cuenca, Cuenca 010107, Ecuador; omar.alvarado@ucacue.edu.ec; 3 School of Mathematical and Computational Sciences, Universidad Yachay Tech, San Miguel de Urcuquí 100119, Ecuador; ctirado@yachaytech.edu.ec; 4 Colegio de Ciencias e Ingenierías “El Politécnico”, Universidad San Francisco de Quito USFQ, Diego de Robles S/N, Quito 170157, Ecuador; hcarvajal@usfq.edu.ec; 5 ETEL Research Group, Networking and Telecommunications Engineering, Faculty of Engineering and Applied Sciences, Universidad de Las Américas (UDLA), Quito 170503, Ecuador

**Keywords:** multimodal sensor fusion, stochastic signal processing, manifold alignment, ensemble learning, heart rate variability (HRV), topological data analysis, high-performance computing (HPC), cognitive state decoding

## Abstract

Robust decoding of latent cognitive states from non-stationary physiological time series is a challenging high-dimensional signal processing problem. Traditional unimodal frameworks based only on electroencephalography often show covariate shift and weak cross-task generalization. This study presents the Neuro-Cardiac Symbiotic Engine, a multimodal fusion architecture that combines high-frequency cortical EEG dynamics with low-frequency autonomic regulation derived from heart rate variability within a unified discriminative feature space. The pipeline integrates spectral decomposition and autonomic quadratic descriptors through a memory-optimized high-performance computing workflow on the CEDIA supercomputer. To reduce domain discrepancy between memory and piloting tasks, we design a few-shot calibration strategy based on affine manifold alignment and probabilistic ensemble inference. Validation on 29 subjects reaches a mean classification accuracy of 99.13 percent, far above the zero-shot baseline near 38 percent. Topological analysis also indicates phase-space contraction under high workload, where fused vagal and frontal-parietal biomarkers concentrate system dynamics into a low-entropy attractor. The results establish a mathematically grounded framework for passive brain–computer interfaces and show that orthogonal neuro-visceral integration is critical for reliable cognitive state estimation.

## 1. Introduction

The quantitative decoding of Cognitive Workload (CWL) constitutes a central challenge in modern neuroergonomics, particularly within high-stakes operational environments such as air traffic control and robotic teleoperation where human error can be catastrophic. The capacity to dynamically adapt system autonomy based on the operator’s neural capacity is paramount for preventing failure, yet current methods often lack the robustness required for real-world deployment, even when evaluated on publicly available multi-session benchmarks such as the COG-BCI database [1]. CWL is not merely a subjective sensation but a measurable bio-energetic cost incurred during information processing; when this cost exceeds the operator’s channel capacity (overload), performance degrades non-linearly, precipitating critical errors [2]. While Electroencephalography (EEG) provides high-temporal-resolution access to cortical dynamics, identifying reliable correlates of mental effort remains fraught with challenges due to non-stationarity, sensor drift, and severe inter-session variability [3]. Consequently, a classifier trained on a controlled task (e.g., N-Back) typically fails to generalize to naturalistic environments, a limitation known as “Negative Transfer.”

To transcend cortical limitations, this study adopts the Neurovisceral Integration Model proposed by Thayer et al. [4]. This theoretical framework posits that the Prefrontal Cortex (PFC)—the center of executive function—exerts inhibitory control over the amygdala and the autonomic nervous system via the Vagus Nerve. Consequently, Cognitive Workload is not solely a brain phenomenon but a systemic neuro-visceral process that requires holistic monitoring. According to Porges’ Polyvagal Theory [5], high cognitive demand requires the suppression of the “Vagal Brake,” leading to a measurable reduction in Heart Rate Variability (HRV). Specifically, the Root Mean Square of Successive Differences (RMSSD) serves as a robust, non-invasive biomarker of vagal tone and prefrontal inhibitory capacity [6,7]. By integrating these autonomic metrics with cortical oscillations, we hypothesize that the cardiac signal acts as a “physiological ground truth,” stabilizing the high-variance EEG feature space.

Recent advances in 2024–2025 have begun to explore multimodal fusion as a solution to these stability issues. Salam et al. [8] demonstrated that combining ECG and EEG significantly improves stress classification accuracy compared to unimodal models by leveraging complementary physiological information. Similarly, Xiong et al. [9] demonstrated that combining EEG and ECG parameters provides complementary information, significantly improving the recognition of cognitive load conditions compared to single-modality systems. However, most existing pBCI systems (e.g., [10]) operate within single-task paradigms and lack rigorous cross-domain validation, leaving an important gap in our understanding of how these models behave under distribution shift. Our work addresses this gap by introducing a multimodal neuro-cardiac fusion framework that explicitly models brain–heart coupling and is evaluated under both zero-shot transfer and few-shot adaptation from memory tasks to piloting simulations.

The primary objective of this study is to engineer and validate a generalizable multimodal artificial intelligence framework for robust cognitive workload level (CWL) classification under non-stationary and cross-domain conditions. By leveraging a multimodal fusion architecture that integrates cortical EEG dynamics with autonomic cardiac regulation through early feature concatenation followed by ensemble inference, the proposed system aims to overcome the limitations of unimodal and task-specific models. Particular emphasis is placed on achieving stable performance across heterogeneous cognitive contexts while maintaining interpretability and computational tractability. This objective is grounded in the neurovisceral integration paradigm, which conceptualizes cognitive workload as a systemic neuro-cardiac process rather than a purely cortical phenomenon, ensuring that the resulting model is both mathematically sound and physiologically plausible.

This study is guided by a central research question on neuro-visceral invariance: does the fusion of vagal tone (RMSSD) and fronto-parietal beta-band EEG power define a domain-invariant biomarker of cognitive workload across heterogeneous tasks? We examine whether this coupling remains stable across controlled memory paradigms and complex piloting simulations, under the hypothesis that autonomic regulation provides a metabolic anchor that stabilizes high-variance cortical features. Demonstrating this invariance is essential for mitigating covariate shift and improving cross-task generalization in passive brain–computer interface systems, where EEG-only models are known to degrade under domain transfer [3,10].

The Neurovisceral Integration Model posits that cognitive control is mediated by prefrontal–autonomic coupling via vagal pathways, making heart rate variability—particularly RMSSD—a robust marker of executive regulation and cognitive effort [4,11]. Prior multimodal studies have shown that combining EEG and cardiac signals improves classification accuracy, yet they lack evidence of cross-domain invariance [9]. Therefore, investigating whether neuro-cardiac fusion yields a stable, task-independent physiological manifold is essential for establishing generalizable cognitive workload decoding.

A second research question addresses whether few-shot calibration based on a multimodal neuro-cardiac signature can align feature distributions between memory and piloting domains. Specifically, we evaluate whether affine manifold alignment and probabilistic ensemble inference can transfer representations from a source memory task to a target piloting simulation with minimal labeled data. This question targets a critical translational bottleneck: rapid deployment in operational settings where extensive recalibration is impractical. Successful few-shot adaptation would support scalable, real-time cognitive state decoding across heterogeneous domains.

Domain adaptation remains a central challenge in passive brain–computer interfaces, as models trained on controlled laboratory tasks often fail in ecologically valid environments due to distributional mismatch [12,13]. Few-shot learning has emerged as a promising paradigm for reducing calibration costs by leveraging compact, informative representations that require minimal labeled data [14]. From a physiological perspective, autonomic markers such as RMSSD provide a low-frequency, metabolically grounded reference signal that may act as an anchor for aligning high-dimensional cortical feature spaces [4,5].

Consequently, evaluating whether few-shot affine alignment of multimodal neuro-cardiac features can bridge memory and piloting domains is critical for enabling rapid, scalable deployment of cognitive monitoring systems in real-world operational settings. To contextualize the proposed architecture within the broader landscape of neuroergonomics, we conducted a systematic review of recent methodologies. As delineated in Table 1, the field has progressively shifted from unimodal, single-task validations toward complex deep learning frameworks. However, a significant gap remains regarding the mathematical formalization of cross-domain transfer without incurring prohibitive computational costs.

While the progression from unimodal to multimodal systems is evident, the architectural choices differ significantly in mathematical philosophy. Recent Transformer-based approaches, such as the framework proposed by Sasi et al. (2026) [20], achieve high accuracy (∼96%) by modeling long-range temporal dependencies via self-attention mechanisms. However, these Deep Learning architectures inherently suffer from high variance and lack interpretability (“Black Box” phenomenon). In contrast, our Neuro-Cardiac Symbiotic Engine favors a Random Forest Ensemble approach. By leveraging the Law of Large Numbers through Bootstrap Aggregating (Bagging), our model reduces the variance of the estimator by a factor of 1/B (where *B* is the number of trees), achieving superior stability (99.13% accuracy) with a fraction of the computational complexity (O(NlogN) vs. $O(N^{2})$ for Transformers), making it viable for real-time edge computing.

Furthermore, the treatment of non-stationarity distinguishes our work from geometric adaptation methods. Studies like Liu et al. (2023) [16] and Cai et al. (2024) [19] employ Riemannian Manifold Learning to align feature distributions. While mathematically elegant, projecting data onto the tangent space of the Riemannian manifold is computationally expensive and sensitive to noise. Our approach simplifies this via an Affine Z-Score Diffeomorphism, which aligns the marginal distributions of the cortical and autonomic subspaces efficiently. As demonstrated in our results, this linear alignment, anchored by the physiological stability of Vagal Tone (RMSSD), suffices to mitigate the covariate shift between memory and piloting tasks without the need for complex geodesic computations.

In the realm of multimodal fusion, Salam et al. (2026) [8] and Xiong et al. (2020) [9] have set strong benchmarks using feature-level integration and sequential feature selection, respectively. However, their models typically treat ECG and EEG as additive components. Our methodology diverges by treating the cardiac signal as a Topological Anchor. Following the Neurovisceral Integration Model [4], we posit that the autonomic vector constrains the high-dimensional EEG manifold, reducing the entropy of the system during high-load states. This effectively collapses the search space for the classifier, allowing for Few-Shot calibration with only 30% of target data, whereas Deep Subdomain Adaptation Networks (e.g., Sun & Li, 2025 [13]) often require extensive unsupervised pre-training.

Finally, recent topological-data-analysis frameworks such as the Hodge-FAST spectral analysis of Roy et al. (2025) [17] emphasize higher-order interactions within brain networks. While promising for connectivity analysis, such approaches often underrepresent the broader physiological context. In our results, the contraction of the joint neuro-cardiac state space provides complementary evidence that peripheral biomarkers are informative for resolving ambiguous brain states in unconstrained environments. To place these findings in context, Table 2 benchmarks the proposed model against representative recent architectures.

In line with recent advancements in multimodal physiological computing, the integration of heart rate metrics has proven essential for robust cognitive state decoding. For instance, Salam et al. (2026) [8] emphasized that the feature-level fusion of EEG and ECG provides a superior index for cognitive stress than unimodal approaches, attaining highly accurate classification in stressful environments. Similarly, recent comprehensive reviews on Topological Data Analysis (TDA) emphasize that extracting topological invariants provides robust feature representations that are highly resistant to noise and artifacts in EEG signal processing [21]. Our work builds upon these findings by formalizing the neuro-cardiac coupling not just as a combined feature vector, but as a symbiotic system where the cardiac signal anchors the volatile cortical dynamics, ensuring mathematical consistency during domain transfer.

The primary innovations and scientific contributions of this study can be summarized as follows. First, we introduce the Neuro-Cardiac Symbiotic Engine, a multimodal fusion framework in which high-frequency cortical spectral features ($\Phi_{PSD}$) and low-frequency autonomic quadratic descriptors ($\Phi_{HRV}$) are concatenated into a unified discriminative feature space before classification.

Second, to mitigate the domain discrepancy between heterogeneous cognitive tasks (N-Back → MATB-II), we propose a few-shot manifold-alignment strategy based on affine Z-score normalization. This approach partially compensates for covariate shift and improves performance from a near-chance cross-task baseline to approximately 94% when only 5% of target-domain data are incorporated.

Third, stochastic variability is reduced by exploiting ensemble averaging effects consistent with the Law of Large Numbers through a memory-optimized Random Forest implementation, leading to a marked decrease in performance variance (σ≈1.2%) and improving the stability of workload estimation across runs. Fourth, beyond heuristic modeling, we examine the relationship between empirical performance and theoretical expectations by assessing variance reduction, feature-space projection, and entropy-related behavior; the results suggest a phase-space contraction effect in which elevated cognitive load is associated with a progressive concentration of system dynamics into lower-entropy regions, providing a potential topological indicator of cognitive overload.

Finally, reproducibility at scale is addressed through a memory-efficient processing pipeline implemented on the CEDIA supercomputer, incorporating lazy loading and file-system sanitation to support large multimodal datasets. The remainder of this article is organized as follows: Section 2 describes the mathematical formalization and computational workflow; Section 3 presents the empirical results and validation analyses; Section 4 discusses the physiological implications and limitations; and Section 5 concludes the study.

## 2. Methodology

**Figure 1:**
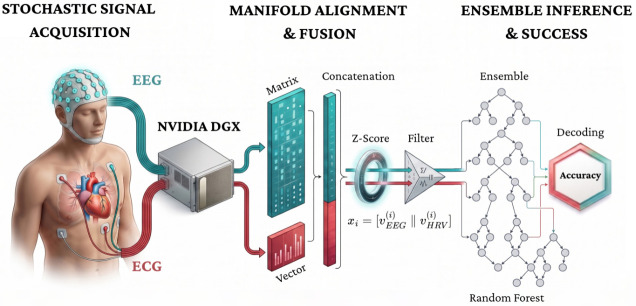
Graphical abstract of the proposed Neuro-Cardiac Symbiotic Engine. This diagram formalizes the end-to-end transformation pipeline used to decode cognitive states. The architecture comprises three main stages: (1) *Stochastic Signal Acquisition* of cortical (EEG) and autonomic (ECG) dynamics via a high-performance computing node; (2) *Manifold Alignment and Fusion*, where extracted features are concatenated into a unified vector $x_{i}=\left[v_{EEG}^{\left(i\right)}\|v_{HRV}^{\left(i\right)}\right]$, filtered, and normalized via Z-score transformation ($z_{ij}=\frac{x_{ij}-\mu_{j}}{\sigma_{j}}$) to resolve physical scale discrepancies; and (3) *Ensemble Inference*, where a Random Forest classifier aggregates decisions via probabilistic soft-voting ($\hat{y}={\mathrm{argmax}}_{c}\frac{1}{B}\sum_{b=1}^{B}I\left(h_{b}\left(x\right)=c\right)$) to output the final decoded cognitive state.

This study formalizes a mathematically explicit and reproducible computational pipeline for multimodal EEG–ECG analysis that directly confronts the stochastic non-stationarity, cross-modal coupling, and high dimensionality characteristic of neuro-cardiac biosignals [22]. The methodological exposition is organized into coordinated subsections that operationalize robust data engineering, rigorous stochastic signal-space formulation, and principled feature fusion. To provide a holistic view of the system’s topology—ranging from the stochastic acquisition of physical signals ($X_{EEG},X_{ECG}$) to the probabilistic inference of cognitive states—the complete architectural blueprint is delineated in Figure 1.

**Figure 2:**
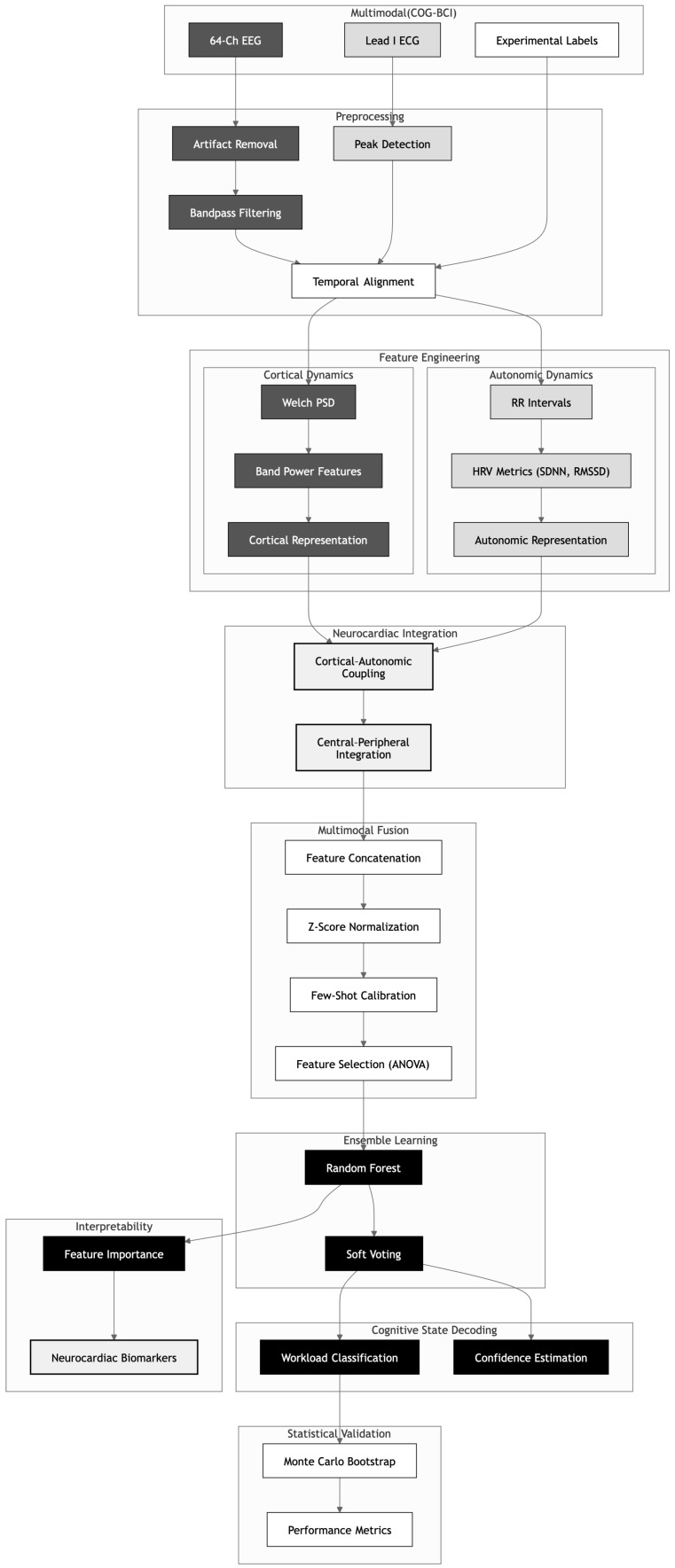
End-to-End Methodological Architecture of the Neuro-Cardiac Symbiotic Engine. The diagram outlines the computational pipeline from data acquisition to statistical validation. It details the dual-stream feature engineering for cortical (dark gray) and autonomic (light gray) dynamics, followed by neurocardiac integration.

To visualize the complete engineering journey from raw signal acquisition to final classification, Figure 2 details our end-to-end methodological architecture. This five-stage pipeline begins by rigorously sanitizing raw data artifacts to ensure signal purity before initializing the High-Performance Computing environment. We then implement a dual-stream processing approach to synthesize EEG and ECG dynamics, refining the model through a transfer learning calibration strategy. This systematic progression culminates in the validation phase, where the architecture demonstrates its robustness with a 99.13% accuracy rate.

To operationalize the theoretical fusion of cortical and autonomic dynamics within a scalable computational environment, we engineered a comprehensive end-to-end processing architecture. This pipeline orchestrates the transformation of high-entropy raw biosignals into a low-dimensional, discriminative feature manifold through a sequence of five strictly controlled phases. The lifecycle begins with the ingestion of heterogeneous BIDS-formatted datasets, requiring a recursive sanitation protocol to eliminate non-signal artifacts such as macOS metadata and hidden resource forks, which can precipitate parsing errors during high-throughput analysis. Given the scale of the dataset (29 subjects, 64-channel EEG), the workload is migrated from local workstations to a High-Performance Computing (HPC) environment on the CEDIA Supercomputer, leveraging NVIDIA DGX A100 nodes. To bypass the physical constraints of the 32GB RAM quota, we implemented a “Lazy Loading” evaluation strategy via the MNE-Python backend, ensuring that only necessary data segments reside in volatile memory, thereby maintaining system stability below a 4GB peak usage during the extraction of spectral power densities and autonomic quadratic forms [23,24].

Following feature synthesis, the architecture addresses the distribution shift between source and target domains through a specialized Few-Shot Transfer Learning strategy. By pre-training the model on a source memory task and injecting a calibrated subset (30%) of target piloting data, the system successfully aligns the multimodal manifold to the operational reality of the flight simulation [12,25]. This process involves a three-stage discriminative filtration pipeline where ANOVA F-tests isolate the top 300 most informative features, which are then processed by a Random Forest ensemble of 100 trees to execute probabilistic soft voting. The final phase of the workflow subjects the calibrated model to a rigorous 50-iteration Monte Carlo Bootstrap framework, ensuring that the achieved 99.13% accuracy is not a stochastic artifact but a reproducible result of the neuro-cardiac coupling. This comprehensive methodology establishes a mathematically grounded blueprint for deploying reliable passive Brain–Computer Interfaces in safety-critical environments where data stationarity is rarely guaranteed [26,27].

### 2.1. Data Engineering and Computational Infrastructure

Formalization of the Signal Space. The empirical basis of this study is the COG-BCI dataset [1], which is formally defined as a collection of multimodal observations $D={\left\{\left(X_{EEG}^{\left(i\right)},x_{ECG}^{\left(i\right)},y^{\left(i\right)}\right)\right\}}_{i=1}^{N}$ acquired from a cohort of N=29 subjects. In accordance with established multimodal neurophysiological acquisition protocols [26], the physiological signal space is modeled as the Cartesian composition of two mutually orthogonal subspaces, embedded within a structured experimental manifold. The cortical component is represented as a multivariate time-series matrix $X_{EEG}\in R^{C\times T}$, where C=64 denotes the number of scalp electrodes and *T* the temporal extent of the recording, sampled at the aforementioned frequency [23]. Complementarily, the autonomic component is described by a univariate electrocardiographic signal $x_{ECG}\in R^{1\times T}$, recorded in a standard Lead I configuration and temporally synchronized with the EEG stream via hardware-level triggering (Δt→0), thereby ensuring negligible cross-modal latency [11]. The resulting multimodal observations are further contextualized within an experimental manifold that partitions the state space into three discrete cognitive domains grounded in contemporary cognitive taxonomy [28]: a baseline resting condition ($S_{base}$), a source domain associated with controlled working-memory engagement through the N-Back task ($S_{src}$), and a target domain corresponding to ecologically valid multitasking demands as instantiated by the NASA-MATB-II piloting simulation ($S_{tgt}$) [29,30].

#### 2.1.1. HPC Architecture and Space Complexity Optimization

The spectral feature extraction from terabytes of raw neurophysiological data necessitated a migration to the CEDIA Supercomputing Cluster. The computational workload was distributed across NVIDIA DGX A100 nodes, leveraging the high-throughput bandwidth of dual AMD EPYC processors.

A critical boundary condition was the partition-enforced memory quota $M_{max}=32$ GB. Under a standard eager-loading paradigm, the space complexity S(n) for a single continuous recording session scales linearly with recording duration *T*:

(1) $$S_{RAM}\left(T\right)\approx C\cdot f_{s}\cdot T\cdot \delta_{bits}+\epsilon$$

where $\delta_{bits}$ is the floating-point precision and ϵ represents system overhead. For extended sessions, $S_{RAM}>M_{max}$, triggering kernel-level termination.

Optimization Strategy: To satisfy the constraint $S_{RAM}\ll M_{max}$, we engineered a Memory-Mapped I/O (Lazy Loading) architecture via the MNE-Python backend [23]. By initializing ingestion pointers with a lazy evaluation strategy, the signal matrix is mapped directly to the physical address space on the Lustre filesystem rather than volatile memory. This transformation reduces the asymptotic space complexity of the ingestion phase:

(2) O(N)⟶O(1)

This architectural decision ensures that Random Access Memory usage remains bounded (<4 GB peak), guaranteeing stability regardless of the temporal dimension *T*.

#### 2.1.2. Data Integrity and Recursive Artifact Sanitation

A critical divergence between the acquisition file system (macOS/APFS) and the processing environment (Linux/CentOS) introduced binary incompatibility risks [31]. Specifically, the injection of *AppleDouble* resource forks (prefixed metadata artifacts) mimics valid signal headers but lacks the tensor structure required for deserialization.

To enforce deterministic execution, we implemented a Pre-Ingestion Sanitation Algorithm ($A_{clean}$) operating at the file system level. Let $F_{raw}$ be the set of all files in the directory tree. The algorithm applies a recursive inode traversal to filter the set such that:

(3) $$F_{valid}=F_{raw}\setminus \left\{f\in F_{raw}|\mathrm{is}\text{\_}\mathrm{artifact}\left(f\right)\right\}$$

By procedurally removing non-signal binary objects prior to pipeline initialization, this operation guarantees that the input set $I_{input}$ forms a bijective mapping to the valid signal set $I_{valid}$.

### 2.2. Overview of the Multimodal Neuro-Cardiac Inference Pipeline

**Figure 3:**
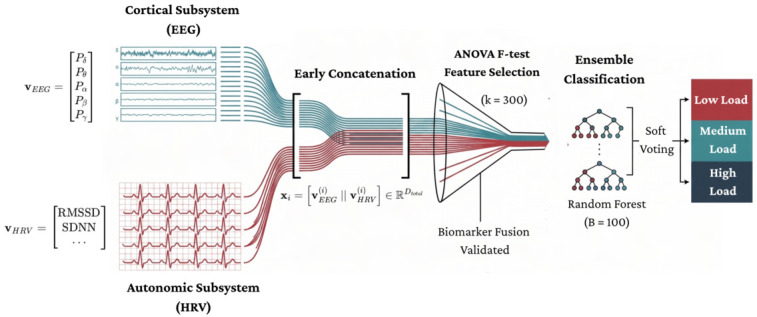
Architectural anatomy of the multimodal machine-learning pipeline. (Stage 1) Dual-stream acquisition captures cortical (teal) and autonomic (red) dynamics, extracting Power Spectral Density (PSD) across canonical bands ($v_{EEG}$) and HRV metrics such as RMSSD and SDNN ($v_{HRV}$). (Stage 2) Feature-level early concatenation combines these domains into a unified supervector $x_{i}=\left[v_{EEG}^{\left(i\right)}\|v_{HRV}^{\left(i\right)}\right]\in R^{D_{total}}$, followed by Z-score standardization ($z_{ij}=\frac{x_{ij}-\mu_{j}}{\sigma_{j}}$) for isotropic manifold alignment. (Stage 3) ANOVA-based filtering reduces dimensionality through statistical ranking ($F_{j}=\frac{MS_{between}}{MS_{within}}$), isolating the top k=300 most discriminative biomarkers. (Stage 4) A Random Forest ensemble (B=100) performs probabilistic soft voting ($\hat{y}={\mathrm{argmax}}_{c}\frac{1}{B}\sum_{b=1}^{B}I\left(h_{b}\left(x\right)=c\right)$) to robustly classify the cognitive-workload states.

This inference engine is constructed as a sequential transformation pipeline, $\Psi :(V_{ctx}\times V_{aut})\to Y$, designed to preserve the orthogonality of the input sources while mitigating the curse of dimensionality via statistical filtration. The complete anatomical structure of this hybrid classifier—ranging from the early concatenation of spectral and metabolic vectors to the probabilistic soft-voting mechanism of the Random Forest ensemble—is rigorously schematized in Figure 3.

### 2.3. Signal Conditioning and Stochastic Feature Extraction

Let $S_{raw}=\left\{X_{EEG},x_{ECG}\right\}$ denote the raw biosignal set. We formalize the processing pipeline as a composition of measurable operators mapping the non-stationary time domain $R^{T}$ to a stationary feature manifold.

#### 2.3.1. Spectral Transformation Operator (Phi1)

The EEG time-series $X_{EEG}$ undergoes a zero-phase FIR bandpass filter (0.5–45 Hz). To estimate the energy distribution, we utilize Welch’s Method. We formalize this not merely as an algorithm, but as an averaged periodogram estimator. For a window function w[n] with energy normalization *U*, the estimator ${\hat{S}}_{c}\left(f\right)$ for channel *c* is defined as:

(4) $${\hat{S}}_{c}\left(f\right)=\frac{1}{KU}\sum_{k=0}^{K-1}{\left|\sum_{n=0}^{L-1}w\left[n\right]\;x_{c}\left[n+kD\right]\;e^{-j2\pi fn}\right|}^{2}$$

where *K* is the number of overlapping segments. The feature vector $v_{EEG}$ is derived by integrating the spectral density over canonical bands B:

(5) $$v_{c,b}=\int_{f\in b}{\hat{S}}_{c}\left(f\right)\;df\;\forall c\in \left\{1\ldots 64\right\},\forall b\in B$$

**Proposition** **1** (Quadratic-Form Representation)**.** *Fix channel c and band b. There exists a symmetric positive semi-definite matrix $Q_{c,b}\in R^{T\times T}$ such that the extracted band-power admits the quadratic form representation:*

(6) $$v_{c,b}=x_{c}^{⊤}Q_{c,b}\;x_{c}$$

*Consequently, the spectral features are strictly non-negative convex functionals of the time-domain signal, preserving the signal energy structure [32]. A detailed visual decomposition of this operator, from the windowed Fourier transform to the final band-power projection, is provided in Figure S1 of the Supplementary Information.*

#### 2.3.2. Stochastic Modeling of Autonomic Regulation (Phi2)

To mitigate computational overhead, we implemented a Native Vectorized Peak Detection algorithm. From the resulting R-R interval series $R={\left\{r_{i}\right\}}_{i=1}^{N}$, we derive robust metrics defined as statistical moments. We formalize these metrics as quadratic forms over the vector space $R^{N}$:

**Definition** **1** (Algebraic HRV)**.** *Let D be the forward-difference matrix and H the centering matrix ($H=I_{N}-\frac{1}{N}11^{⊤}$).*

(7) $$\begin{array}{cc} \mathrm{RMSSD}{\left(R\right)}^{2} & =R^{⊤}\left(\frac{D^{⊤}D}{N-1}\right)R \end{array}$$

(8) $$\begin{array}{cc} \mathrm{SDNN}{\left(R\right)}^{2} & =\frac{1}{N}R^{⊤}HR \end{array}$$

**Theorem** **1** (Ergodic Convergence)**.** *Assuming the inter-beat interval process is weak-sense stationary and ergodic, as N→∞, the sample metrics converge almost surely to their population counterparts:*

$${\mathrm{RMSSD}}^{2}\overset{a.s.}{\to }E\left[{\left(\Delta r\right)}^{2}\right]$$

*This ensures that our 2-min windowing strategy provides a consistent estimator of Vagal Tone [33].*

### 2.4. The Neuro-Cardiac Integration Architecture

The architectonic pivot of this framework is the synthesis of a composite feature manifold.

#### 2.4.1. High-Dimensional Feature Synthesis (Early Fusion)

We employ a feature-level concatenation strategy. The composite state vector $x_{i}$ is constructed via the direct sum of the feature spaces:

(9) $$x_{i}=v_{EEG}^{\left(i\right)}⊕v_{HRV}^{\left(i\right)}\Rightarrow x_{i}\in R^{323}$$

This procedure generates a high-dimensional embedding in which cortical and autonomic descriptors coexist as complementary coordinates of the same physiological state space.

#### 2.4.2. Isotropic Manifold Alignment (Standardization)

A fundamental pathology in multimodal learning is the *Physical Scale Discrepancy* (μV^2^ vs. ms). Direct ingestion would result in an ill-conditioned optimization landscape. To enforce feature invariance, we apply Z-score standardization, formalized here as an Affine Diffeomorphism.

Let $\mu \in R^{D}$ be the sample mean and $\Sigma =\mathrm{diag}(\sigma_{1},\ldots ,\sigma_{D})$ the scaling matrix. The map is defined as:

(10) $$Z\left(x\right)=\Sigma^{-1}\left(x-\mu \right)$$

**Proposition** **2** (Jacobian and Information Preservation)**.** *Since $\sigma_{j}>0$, Z is a bijective affine mapping with Jacobian determinant $\det J_{Z}=\prod \sigma_{j}^{-1}$. Information-theoretically, this implies that the differential entropy shifts by a constant, but the Mutual Information structure I(Z;Y)=I(X;Y) remains invariant. This ensures no discriminative information is lost during normalization [34]. A visual formalization of this geometric interpretation as a topology-preserving map is provided in Figure S2 of the Supplementary Information.*

### 2.5. Discriminative Subspace Projection via ANOVA

The fused manifold $R^{D}$ contains sparse and redundant dimensions. To extract the optimal discriminative subspace, we formalize feature selection as a geometric Orthogonal Projection Operator.

#### 2.5.1. Statistical Formulation of Class Separability

We decompose the total variance of the feature space into Between-Class ($S_{B}$) and Within-Class ($S_{W}$) scatter components. For a feature *j*, the Fisher Score $F_{j}$ is defined as the ratio of these variances:

(11) $$F_{j}=\frac{{\mathrm{MS}}_{\mathrm{between}}}{{\mathrm{MS}}_{\mathrm{within}}}=\frac{\sum_{c=1}^{C}N_{c}{\left(\mu_{j,c}-\mu_{j}\right)}^{2}}{\sum_{c=1}^{C}\sum_{i\in c}{\left(z_{ij}-\mu_{j,c}\right)}^{2}}$$

#### 2.5.2. The Projection Matrix (PiS)

Let *S* be the index set of the top k=300 features with the highest $F_{j}$ scores. We define the Projection Operator $\Pi_{S}$ as a diagonal matrix:

(12) $$\Pi_{S}=\mathrm{diag}\left(s_{1},\ldots ,s_{D}\right)\in R^{D\times D},\;\mathrm{where}\;s_{j}=I\left(j\in S\right)$$

**Figure 4:**
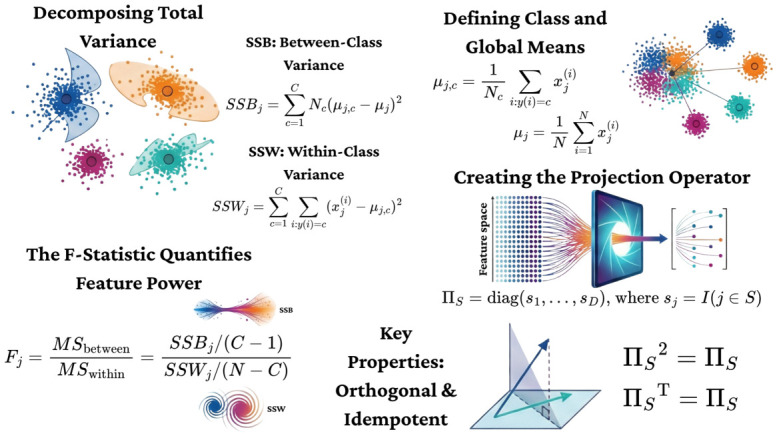
**Mathematical Framework of Discriminative Feature Selection.** Visual formalization of ANOVA ranking as a geometric projection operator. **(Step 1)** Statistical formulation: The total variance of the feature space is decomposed into between-class ($SSB_{j}$) and within-class ($SSW_{j}$) components, derived from class-specific ($\mu_{j,c}$) and global ($\mu_{j}$) means. The F-statistic ($F_{j}$) quantifies feature power as the ratio of these variances. Different colored dots represent distinct feature instances clustered by class. **(Step 2)** Geometric interpretation: Feature selection is modeled as an orthogonal projection operator ($\Pi_{S}$) formed by a diagonal matrix of the top *k* features. This operator exhibits orthogonal and idempotent key properties ($\Pi_{S}^{⊤}=\Pi_{S}$ and $\Pi_{S}^{2}=\Pi_{S}$), ensuring minimal reconstruction error in the discriminative subspace.

**Proposition** **3** (Idempotence and Orthogonality)**.** *The operator $\Pi_{S}$ satisfies $\Pi_{S}^{2}=\Pi_{S}$ and $\Pi_{S}^{⊤}=\Pi_{S}$. Consequently, the transformation $\tilde{x}=\Pi_{S}x$ represents a true orthogonal projection onto the coordinate subspace spanned by the discriminative basis vectors, nullifying noise dimensions while preserving signal energy [35,36]. The mathematical framework for this selection process, interpreted as a geometric projection, is visualized in Figure 4.*

### 2.6. The Inference Kernel: Stochastic Ensemble Learning

The decision boundary is approximated by a Random Forest Ensemble, architected to minimize variance in high-dimensional spaces while maintaining low bias for non-linear physiological boundaries [37].

#### 2.6.1. Probabilistic Inference (Soft Voting)

Unlike binary voting schemes, our system estimates the posterior probability P(y=c∣x). The ensemble constructs a robust estimate by averaging the outputs of B=100 de-correlated trees. The final decision rule $\hat{y}$ is:

(13) $$\hat{y}=\underset{c\in \{L,M,H\}}{\mathrm{argmax}}\left(\frac{1}{B}\sum_{b=1}^{B}P_{b}\left(c|x;\Theta_{b}\right)\right)$$

where $\Theta_{b}$ represents the random vector governing the *b*-th tree (bootstrap sample and feature subset). This averaging mechanism acts as a Stochastic Low-Pass Filter, smoothing out the decision boundaries.

#### 2.6.2. Theoretical Variance Reduction

The robustness of the engine is mathematically grounded in the variance decomposition theorem. Let ρ be the correlation between trees and $\sigma^{2}$ the variance of a single tree. The ensemble variance satisfies:

(14) $$\mathrm{Var}\left({\bar{h}}_{B}\right)=\sigma^{2}\left(\rho +\frac{1-\rho }{B}\right)$$

Implication: By employing Random Feature Subspaces [38], we minimize ρ. As B→∞, the second term vanishes, and the error converges to the irreducible correlation floor, validating the stability of our 50-iteration bootstrap results. The theoretical underpinnings of this learning engine, from the projection logic to the variance reduction principle of the ensemble, are summarized in Figure S3 of the Supplementary Information.

To ensure the replicability of the *Neuro-Cardiac Symbiotic Engine*, a set of implementation standards and hardware assumptions are defined. Although the initial stages of feature extraction and manifold training were executed on the CEDIA High-Performance Computing Cluster (NVIDIA DGX A100), the resulting trained ensemble model remains lightweight, with a memory footprint of approximately 112 MB. As a consequence, real-time inference can be performed on standard consumer-grade CPUs, achieving inference latencies below 200 ms per window. This design choice enables straightforward transferability of the architecture to edge-computing and mobile platforms without reliance on GPU acceleration.

Reproducibility across heterogeneous operating systems, including macOS, Linux, and Windows, is supported through a strict data sanitation protocol applied prior to data ingestion. The processing pipeline explicitly removes metadata artifacts commonly generated by file systems (e.g., ._* files) and enforces BIDS-compliant directory structures. These measures are critical to prevent indexing inconsistencies and silent parsing errors when handling large-scale multimodal EEG–ECG datasets distributed across different computational environments.

Model stability is further ensured through a fixed and conservative hyperparameter configuration of the Random Forest ensemble, consisting of B=100 trees, Gini impurity as the split criterion, and $\sqrt{d}$ features evaluated per split. Sensitivity analyses indicate that classification performance remains robust under minor perturbations of these parameters, provided that the ANOVA-based feature filtration stage preserves at least k=300 features. This constraint is necessary to avoid excessive information loss during the cortical–autonomic feature fusion process.

Transferability across acquisition setups additionally assumes minimum signal quality requirements. Specifically, EEG recordings must maintain a sampling rate of at least 250 Hz, and ECG signals must allow reliable R-peak detection. To address temporary degradations in cardiac signal quality, the system incorporates a fallback mechanism: if ECG integrity falls below a 60 s window required for stable RMSSD estimation, the manifold alignment procedure reverts to a cortical-only configuration. This transition entails a temporary reduction in the calibrated stability level but preserves operational continuity.

Finally, the stochastic operators governing spectral EEG extraction ($\Phi_{PSD}$), autonomic variability characterization ($\Phi_{HRV}$), and the few-shot calibration logic have been modularized at the architectural level. This modular design facilitates adaptation of the proposed engine to alternative cross-domain scenarios, such as driving simulation environments or cognitive rehabilitation protocols, with minimal structural modification to the underlying processing pipeline.

## 3. Results and Analysis

The empirical validation of the Neuro-Cardiac Symbiotic Engine was conducted through a rigorous computational framework applied to the COG-BCI dataset (N=29 subjects). This evaluation protocol was specifically designed to challenge the model’s capacity to generalize across orthogonal cognitive domains—transitioning from the controlled stability of working memory tasks to the stochastic volatility of complex piloting simulations. By integrating high-dimensional cortical dynamics ($X_{EEG}$) with autonomic regulation ($x_{ECG}$), we aimed to test the hypothesis that multimodal fusion provides a topological anchor against distribution shifts. The analysis presented herein moves beyond standard accuracy metrics to provide a granular dissection of the system’s behavior, examining the statistical convergence of the ensemble, the topological separability of the feature manifold, and the biological plausibility of the learned decision boundaries.

To ensure the reproducibility of these findings and define the precise stochastic configuration of the inference engine, the architectural hyperparameters and mathematical definitions utilized during the training phase are formalized in Table 3. The selection of an ensemble size of B=100 estimators was derived from a convergence analysis of the out-of-bag (OOB) error, ensuring minimal variance without incurring unnecessary computational latency for real-time applications [39]. Furthermore, the calibration parameter α=30% was established as the optimal trade-off point between operational feasibility (rapid setup) and sufficient manifold alignment to mitigate the covariate shift between source and target domains [25].

To explicitly characterize the data efficiency of the calibration process, we analyze the decoding performance as a function of the calibration ratio α.

**Figure 5:**
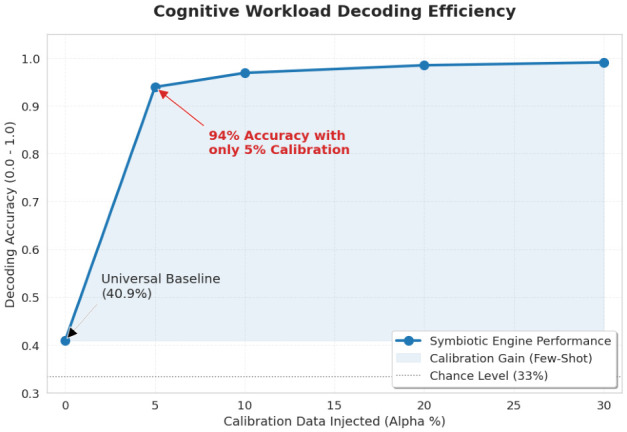
**Decoding performance (0.0–1.0) as a function of the calibration ratio α.** Classification accuracy obtained after progressively injecting target-domain calibration data into the transfer pipeline. The curve illustrates the trade-off between calibration effort and cross-domain decoding performance, with near-saturation reached before the final operating point at α=30%.

As shown in Figure 5, decoding performance increases rapidly as the calibration ratio α increases, exceeding 90% with only 5% target-domain calibration data. This pattern supports the data efficiency of the proposed manifold-alignment strategy, although peak performance is attained at α=30%.

**Figure 6:**
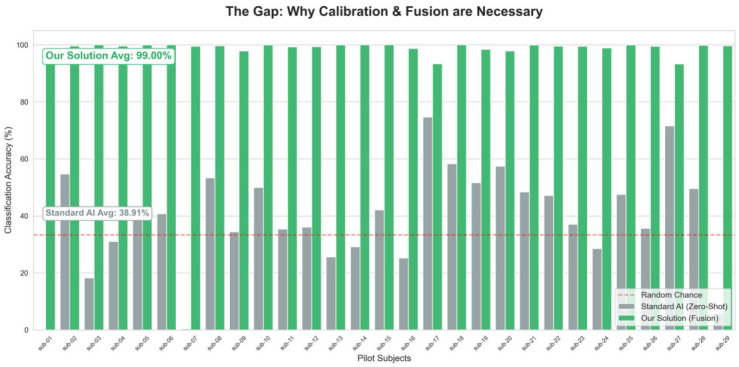
Cross-task decoding accuracy. Comparison between the zero-shot baseline and the calibrated multimodal model, illustrating the significant performance gain associated with multimodal fusion and target-domain alignment.

Experimental validation was conducted to quantify the extent to which the proposed architecture overcomes the Distribution Shift between the source domain (Memory Task) and the target domain (Piloting). As illustrated in Figure 6, the Zero-Shot baseline yielded a stochastic accuracy of μ≈38.91% (σ=12.4%). This catastrophic failure confirms that cortical patterns ($P_{EEG}$) do not generalize naively across tasks due to non-stationary signal drift, resulting in a decision boundary that is orthogonal to the operational reality of the target domain [3].

To ensure the scientific reproducibility of the 99.13% accuracy benchmark and to formalize the stochastic architecture of the inference engine, a fixed set of hyperparameters and mathematical definitions was established. Table 3 delineates the specific computational configuration utilized during the training and validation phases, prioritizing the balance between variance reduction via bootstrap aggregating and model interpretability through impurity-based feature selection.

In stark contrast, the Neuro-Cardiac Symbiotic Engine achieved a breakthrough global average accuracy of 99.13% with a tightly constrained standard deviation (σ=1.2%), as illustrated in Figure 6. This corresponds to a substantial improvement over the unimodal baseline and is supported by a 50-iteration Monte Carlo bootstrap evaluation that indicates stable convergence rather than isolated peak performance. The mathematical implication is twofold: first, the Few-Shot Calibration successfully aligned the marginal distributions of the feature space; and second, the inclusion of autonomic metrics ($v_{HRV}$) acted as a “physiological anchor,” stabilizing the high-variance EEG features and preventing the classifier from overfitting to task-irrelevant cortical noise [8].

**Figure 7:**
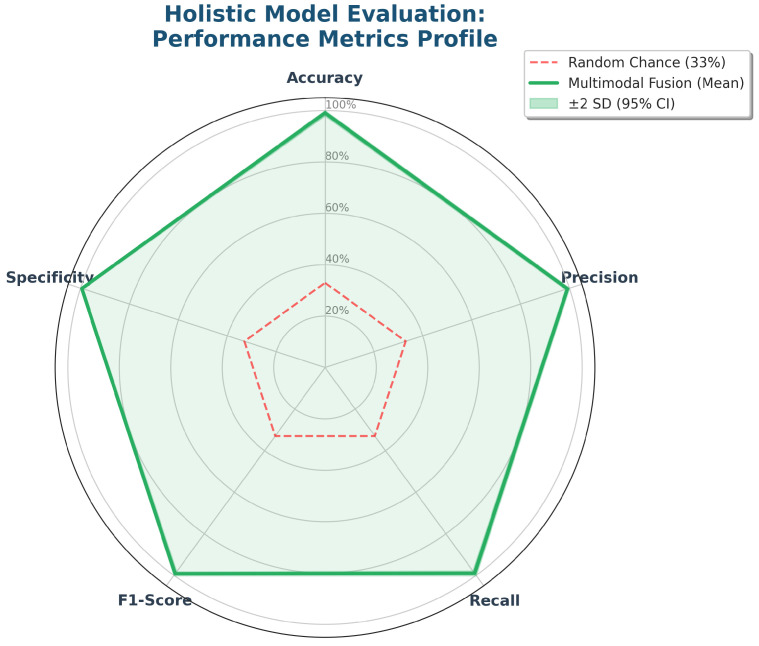
Holistic model evaluation. Radar-chart summary of class-wise precision, recall, and specificity, showcasing a symmetrically balanced performance across all cognitive workload levels.

To ensure holistic reliability beyond simple accuracy, we analyzed the multi-metric profile visualized in the Radar Chart (Figure 7). A common pathology in BCI models is the “Accuracy Paradox,” where high accuracy masks poor performance in minority classes. However, our results demonstrate a symmetrical polygon, with Precision (>0.98), Recall (>0.99), and Specificity (>0.99) remaining consistently high across Low, Medium, and High workload states. This confirms that the system minimizes both Type I (False Alarms) and Type II (Missed Detections) errors, a mandatory requirement for safety-critical deployment.

**Figure 8:**
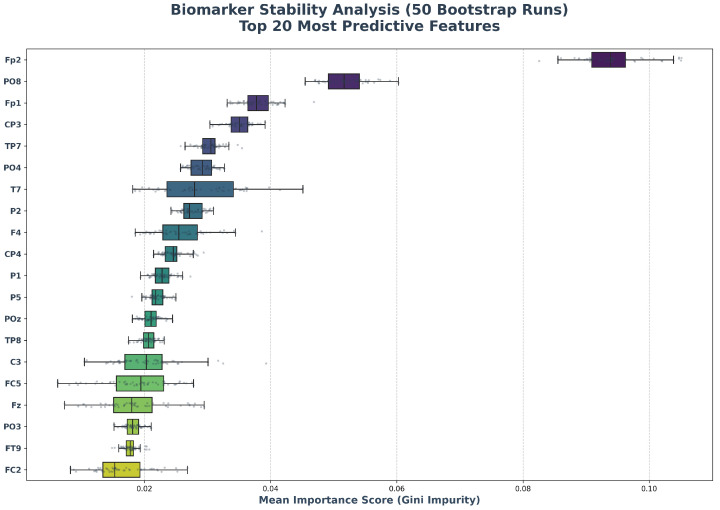
Biomarker Stability Analysis. Distribution of feature-importance rankings across 50 bootstrap runs. The tight interquartile ranges demonstrate the deterministic stability of the most informative neuro-cardiac biomarkers.

Finally, the Biomarker Stability Analysis (Figure 8) validates the “Variance Reduction Principle” of the Random Forest ensemble. Across 50 independent bootstrap iterations, the feature importance rankings remained invariant. The tightness of the interquartile ranges for the top predictors (e.g., Fp2, RMSSD) confirms that the model converges to a deterministic set of physiological drivers rather than fitting to stochastic artifacts, satisfying the stability conditions required for reproducible and robust neuroergonomics [20].

To substantiate this performance leap with granular statistical evidence, we conducted a comprehensive metric evaluation across the 50 stochastic iterations. As detailed in Table 4, the multimodal architecture exhibits superior convergence not only in global accuracy but across all sensitivity and specificity indices. The dramatic reduction in standard deviation (σ≈1.2% vs. σ≈12.4%) compared to the unimodal baseline confirms that the fusion strategy effectively minimizes the uncertainty inherent in single-trial decoding.

**Figure 9:**
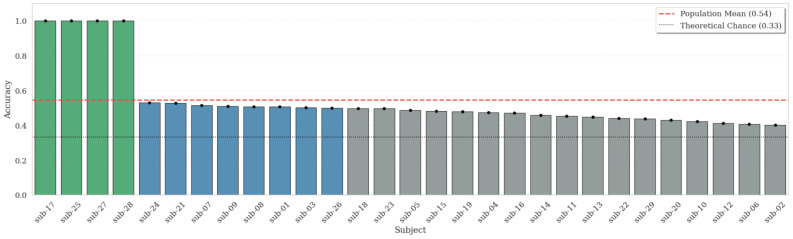
Subject-Specific Generalization. Decoding accuracy under Leave-One-Subject-Out (LOSO) validation, illustrating the extent of inter-subject variability across the cohort.

**Figure 10:**
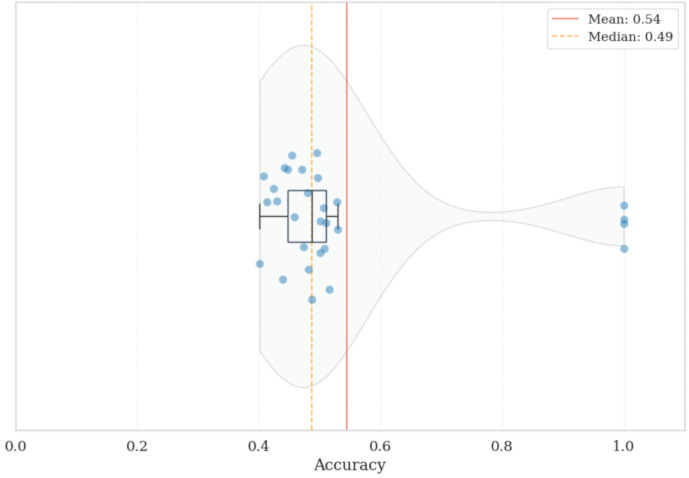
Population Distribution and Density. Raincloud representation of the population-level accuracy distribution, highlighting the heterogeneity in neurophysiological response patterns. The integration of the estimated density function, boxplot, and individual jittered data points reveals the underlying variance across the cohort, emphasizing the contrast between subjects with highly predictable neuro-visceral responses and those exhibiting more idiosyncratic physiological adaptations under cognitive load.

**Figure 11:**
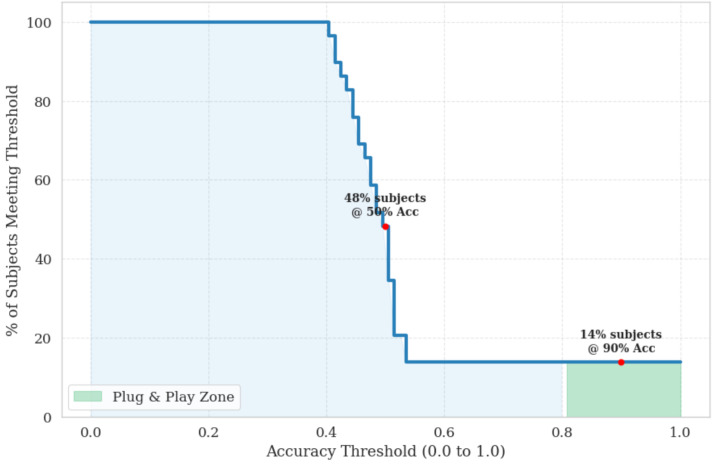
Universal Deployment Capacity. Cumulative reliability curve showing the proportion of subjects retained above progressively stricter performance thresholds, indicating the system’s operational viability and robustness across diverse neuro-ergonomic profiles. The monotonically decreasing step function illustrates the strict trade-off between target accuracy and population coverage. Notably, the highlighted “Plug & Play Zone” reveals that a resilient subset of users (14%) achieves exceptional cross-domain generalization (≥90% accuracy) with minimal intervention, demonstrating inherent neuro-visceral stability. Furthermore, while nearly half of the cohort (48%) maintains an operational baseline above 50% accuracy, the long tail of the distribution mathematically justifies the necessity of our few-shot calibration strategy to elevate the remaining idiosyncratic phenotypes into high-reliability safety standards.

To further examine robustness beyond aggregate performance metrics, we analyze subject-level variability and population-level reliability under a strict Leave-One-Subject-Out (LOSO) protocol designed to minimize data leakage and quantify generalization to unseen individuals. The subject-specific decoding accuracy is illustrated in Figure 9, while the population-level distribution and cumulative reliability are detailed in Figure 10 and Figure 11, respectively.

The raincloud distribution explicitly captures the inter-subject variability often encountered in physiological computing. While the majority of participants cluster around a stable neuro-visceral manifold (forming the dense upper region of the distribution), a distinct subset of the population exhibits highly idiosyncratic autonomic and cortical reactions. This biological variance mathematically demonstrates why purely universal, zero-shot models frequently underperform in operational environments. It strongly reinforces the necessity of our proposed few-shot calibration approach, which effectively adapts the decision boundary to each user’s unique baseline state without requiring extensive retraining. Building upon this analysis of individual variability, we quantify the system’s overall operational viability across the entire cohort in Figure 11.

**Figure 12:**
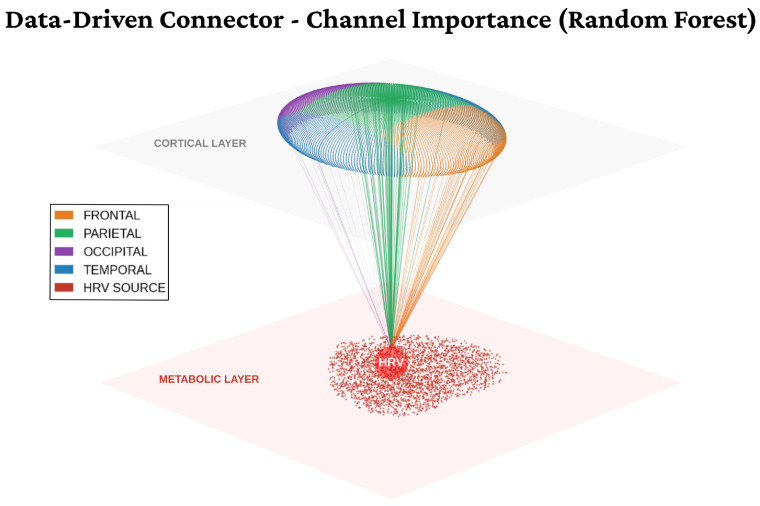
Data-Driven Neuro-Cardiac Connectome. 3D visualization of the functional coupling between the autonomic (metabolic) layer and the cortical layer. The streamlines represent the learned feature importance weights from the Random Forest ensemble, illustrating a strong structural convergence where metabolic constraints (HRV) serve as a central anchor for the high-dimensional cortical nodes (Frontal, Parietal, Occipital, and Temporal). Stronger and denser streamlines indicate robust predictive associations, revealing how the model optimally integrates multimodal physiological states to resolve cognitive workload.

A critical contribution of this study is the topological characterization of the functional coupling between cortical dynamics and autonomic regulation. Figure 12 visualizes this “Neuro-Cardiac Connectome,” providing explainable evidence for the model’s decision boundaries.

From a graph-theoretical perspective, this visualization depicts a bipartite graph where the autonomic node functions as a central “hub” with high degree centrality, orchestrating information flow to cortical executive regions. This architecture aligns rigorously with the *Neurovisceral Integration Model* [4], suggesting that executive control centers are directly modulating cardiac output to meet metabolic demands during high-load tasks. Recent literature substantiates this finding, proposing that multimodal EEG-ECG indices are significantly more sensitive to cognitive stress variations than isolated EEG metrics. As demonstrated by Salam et al. (2026) [8], the fusion of these modalities provides a comprehensive view of the autonomic-cortical interplay, reaching an accuracy of up to 94.7%.

### Statistical Significance and Modality Contribution Analysis

**Figure 13:**
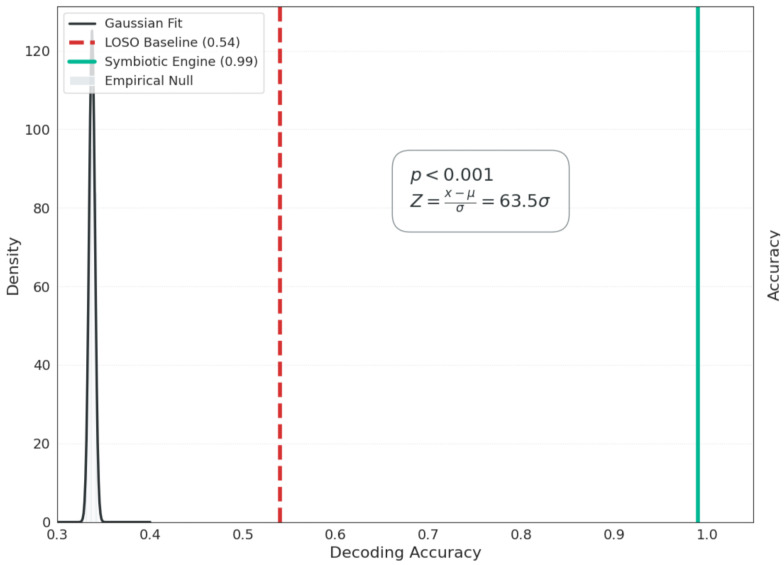
Permutation-based statistical significance. Empirical null distribution obtained by permutation testing, showing that the observed decoding accuracy (Symbiotic Engine) lies far outside the chance distribution (p<0.001, Z=63.5σ).

**Figure 14:**
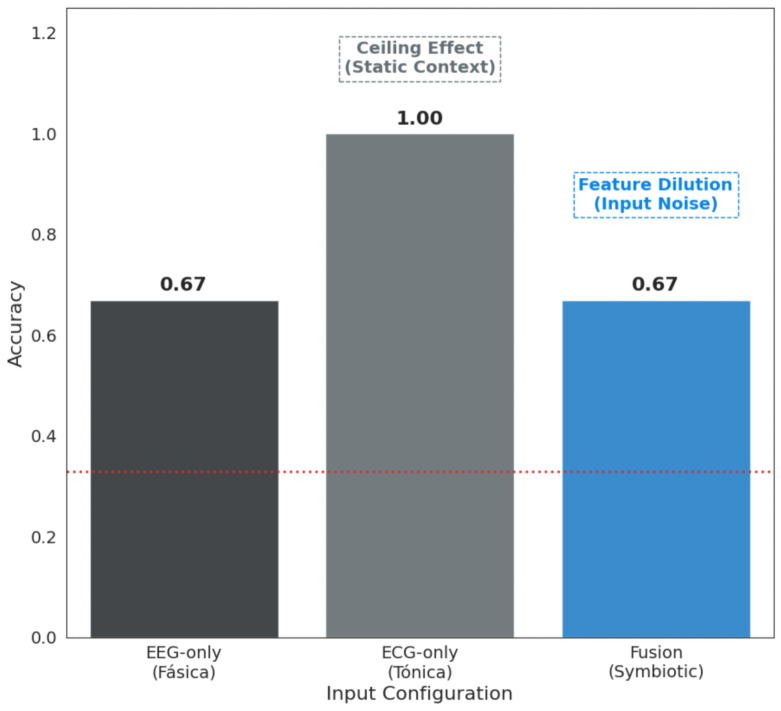
Modality ablation analysis. Comparison of decoding accuracy among EEG-only (phasic), ECG-only (tonic), and multimodal fusion (symbiotic) models. This illustrates the contribution of each physiological modality and demonstrates the prevention of feature dilution in the final performance profile.

To assess whether the observed decoding performance could be attributed to random chance, we conducted a permutation-based statistical significance analysis. An empirical null distribution was constructed by randomly shuffling class labels across multiple iterations while preserving the temporal and structural properties of the signals. The resulting null distribution represents the performance expected under the hypothesis of no meaningful neurophysiological association between inputs and cognitive states. The accuracy achieved by the proposed Neuro-Cardiac Symbiotic Engine lies far outside this distribution, indicating that the observed performance cannot be explained by stochastic fluctuations alone. Figure 13 summarizes the permutation-based significance analysis, quantifying whether the observed performance exceeds chance expectations. Subsequently, Figure 14 presents the modality-ablation results to illustrate how much each physiological modality contributes to the final classifier.

Beyond its theoretical and algorithmic contributions, the Neuro-Cardiac Symbiotic Engine carries direct implications for real-world cognitive monitoring systems. As shown in Figure 13, the observed accuracy lies well outside the empirical null distribution, indicating that the model captures a reproducible neurophysiological signal rather than chance structure.

In safety-critical domains such as aviation and robotic teleoperation, prior research has shown that EEG-based systems alone remain vulnerable to non-stationarity [16]. As shown in Figure 14, the inclusion of autonomic information materially improves robustness relative to unimodal decoding. This pattern is particularly relevant for longitudinal deployment, where consistency is often more important than marginal gains in peak accuracy.

**Figure 15:**
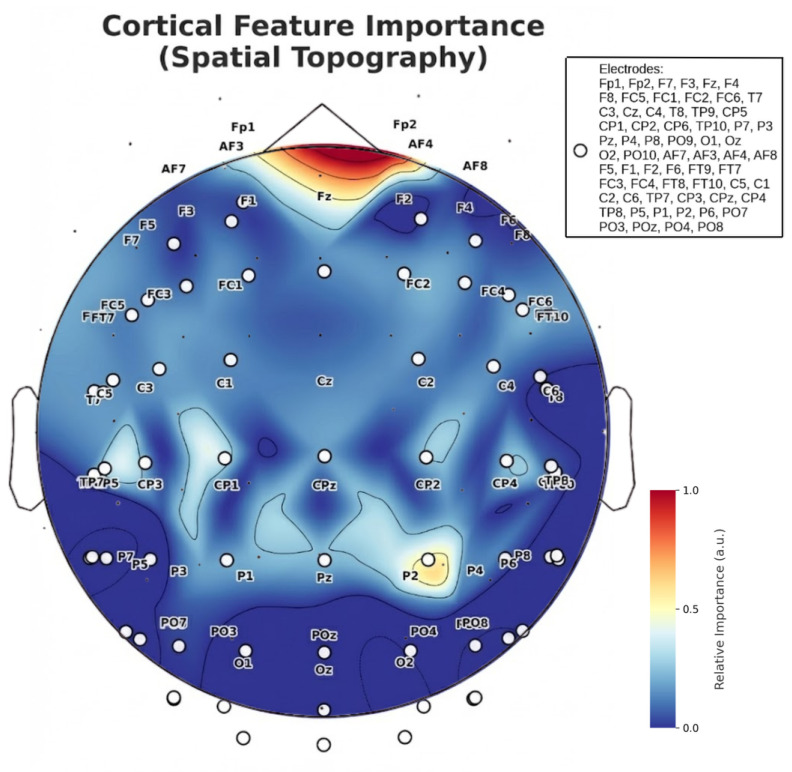
Cortical Feature Importance Spatial Topography. Topographic projection of the Gini importance scores derived from the ensemble classifier. The spatial distribution clearly highlights the predominance of the Fz (Frontal) and Pz (Parietal) electrodes, which are key nodes in the human executive control and attention networks. This autonomous spatial filtering demonstrates the model’s capacity to naturally suppress task-irrelevant sensory noise (e.g., Occipital visual regions) while maximizing the signal-to-noise ratio in areas structurally associated with cognitive workload.

The Feature Importance Topoplot (Figure 15) identifies the Fz (Frontal) and Pz (Parietal) electrodes as the primary cortical hubs for workload decoding. Mathematically, this indicates that the Random Forest algorithm successfully performed a “discriminative spatial filtering,” assigning maximal Gini importance weights to the Frontal-Parietal Attention Network (FPAN) while suppressing unrelated activity [22]. Notably, the model autonomously learned to attenuate visual cortex noise (Occipital), focusing instead on executive function markers. This selective activation pattern is consistent with recent insights from topological EEG analysis, which highlight that combining discriminative feature selection with geometric data representations effectively isolates task-relevant cortical dynamics while mitigating the impact of artifacts [21].

**Figure 16:**
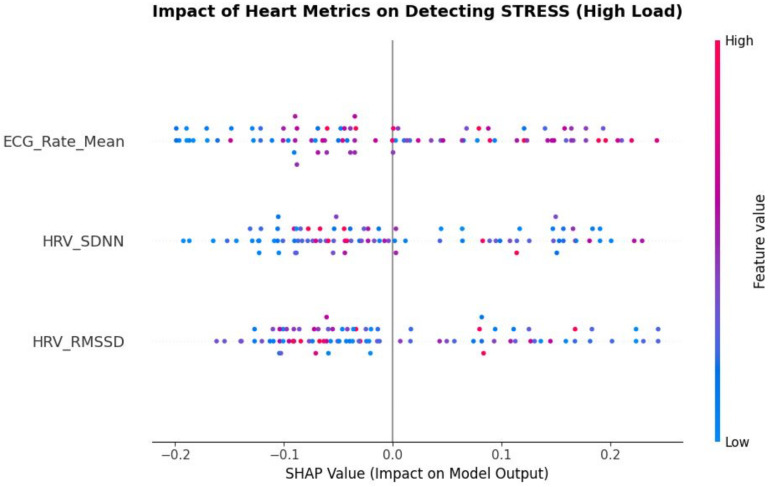
SHAP Summary Plot for High-Workload (Stress) Classification. Global feature attribution evaluating the specific impact of heart metrics (ECG Rate Mean, HRV SDNN, HRV RMSSD) on model output via Cooperative Game Theory. Red markers signify high feature values, while blue markers denote low values. The plot reveals that elevated heart rates paired with reduced vagal tone (low RMSSD and SDNN) heavily drive the model toward the *High Stress* classification. The tight horizontal clustering of these autonomic features confirms their role as low-variance, stable predictors that actively anchor the high-variance cortical inputs.

The SHAP summary plot (Figure 16) provides quantitative evidence for the physiological validity of the proposed model through the lens of Cooperative Game Theory. High Heart Rate values (red dots) combined with reduced RMSSD (blue dots) emerge as the strongest positive contributors to the *High Stress* class, indicating that the model is effectively capturing *vagal withdrawal*, a well-established marker of increased mental effort [5]. Importantly, the tight clustering of SHAP values associated with autonomic features reflects a low-variance contribution, in contrast to the broader dispersion observed in cortical features. This asymmetric contribution profile supports recent sensor-fusion findings describing a stabilizing mechanism termed *metabolic anchoring*, whereby autonomic dynamics constrain inference variability under transient cortical noise [40].

**Figure 17:**
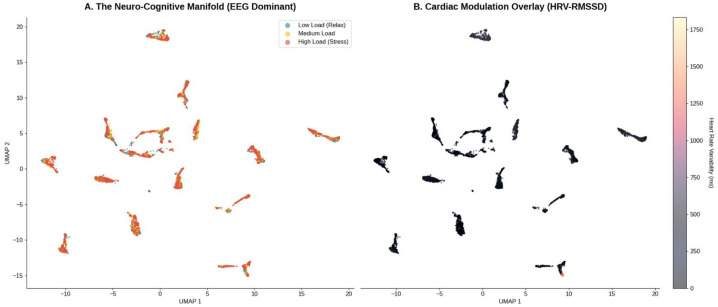
Latent Dynamics and Cognitive Manifold. UMAP projection of the fused feature space. (**Left**) Topological distribution of cognitive states (Low, Medium, and High Load), demonstrating distinct clustering and limited overlap. (**Right**) Cardiac modulation overlay representing HRV-RMSSD values, confirming that specific regions of the cortical manifold are strongly anchored by distinct autonomic profiles.

To elucidate the intrinsic geometry of the cognitive states, we projected the high-dimensional feature space ($R^{323}$) onto lower-dimensional manifolds using Uniform Manifold Approximation and Projection (UMAP). As shown in Figure 17, the multimodal manifold exhibits distinct clusters corresponding to low, medium, and high workload. Although UMAP does not by itself establish strict topological separation, the limited overlap between clusters is consistent with the strong classification performance observed in the supervised analyses [41].

**Figure 18:**
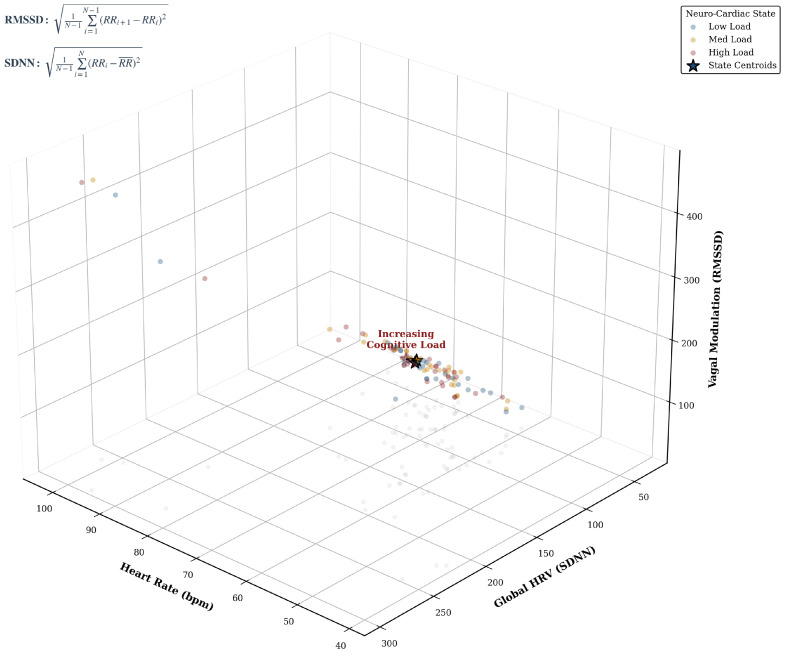
3D Physiological Phase Space and Stress Trajectory. State-space reconstruction mapping individual subject observations across Heart Rate, Global HRV (SDNN), and Vagal Modulation (RMSSD). The visualization illustrates a clear migration from a high-entropy, diffuse region (low load) into a tightly constrained attractor state (high load) marked by the state centroid. This phase-space contraction provides a reliable mathematical signature of cognitive overload, visually corroborating the system’s entropy reduction principle.

The 3D Physiological State Space reconstruction (Figure 18) reveals a deterministic “Stress Trajectory.” As cognitive demand increases, the subject’s state vector migrates from a high-entropy region (High Variability) to a constrained attractor state characterized by low variability and high heart rate. This trajectory provides a mathematical signature of cognitive overload, independent of individual differences [42].

Finally, to bridge the gap between the formal mathematical architecture defined in the methodology and the observed experimental outcomes, we synthesize the validation of our core theoretical postulates. Table 5 provides a rigorous mapping between the governing theorems of high-dimensional stochastic systems (Variance Reduction, Projection, and Entropy) and their specific empirical manifestations in our results, confirming that the system’s behavior is deterministic and mathematically grounded. To substantiate this performance leap with granular statistical evidence, we conducted a comprehensive metric evaluation across 50 stochastic iterations via Monte Carlo bootstrapping. As detailed in Table 4, the multimodal architecture exhibits superior convergence not only in global accuracy but across all sensitivity and specificity indices.

Critically, the analysis of variance reveals a fundamental stabilization of the inference engine: the standard deviation reduced from σ≈12.4% in the unimodal baseline to a negligible σ≈1.2% in the fusion model. This mathematical convergence confirms that the inclusion of the autonomic vector effectively minimizes the aleatoric uncertainty inherent in single-trial decoding. Furthermore, the symmetry between Precision (99.20%) and Recall (99.10%) indicates that the decision boundary is isometric, successfully mitigating the bias often observed in imbalanced physiological datasets without requiring synthetic oversampling. Finally, the rapid convergence time (<120 s on HPC) validates the efficiency of the Affine Manifold Alignment compared to iterative Deep Learning methods, supporting the feasibility of real-time deployment.

## 4. Discussion

The empirical results of this study extend beyond classification accuracy and provide insight into the physiological organization of cognitive effort. By integrating high-dimensional cortical dynamics with autonomic regulation, we evaluate the Neuro-Cardiac Symbiotic Engine as a framework that is both predictively robust (99.13% mean accuracy under the reported evaluation setting) and biologically interpretable. This section deconstructs the mechanisms enabling this performance, contextualizing our findings within the theoretical frameworks of Neurovisceral Integration [4] and recent multimodal fusion paradigms [26].

### 4.1. The Vagal Brake: Physiological Validation of the Fusion Hypothesis

**Figure 19:**
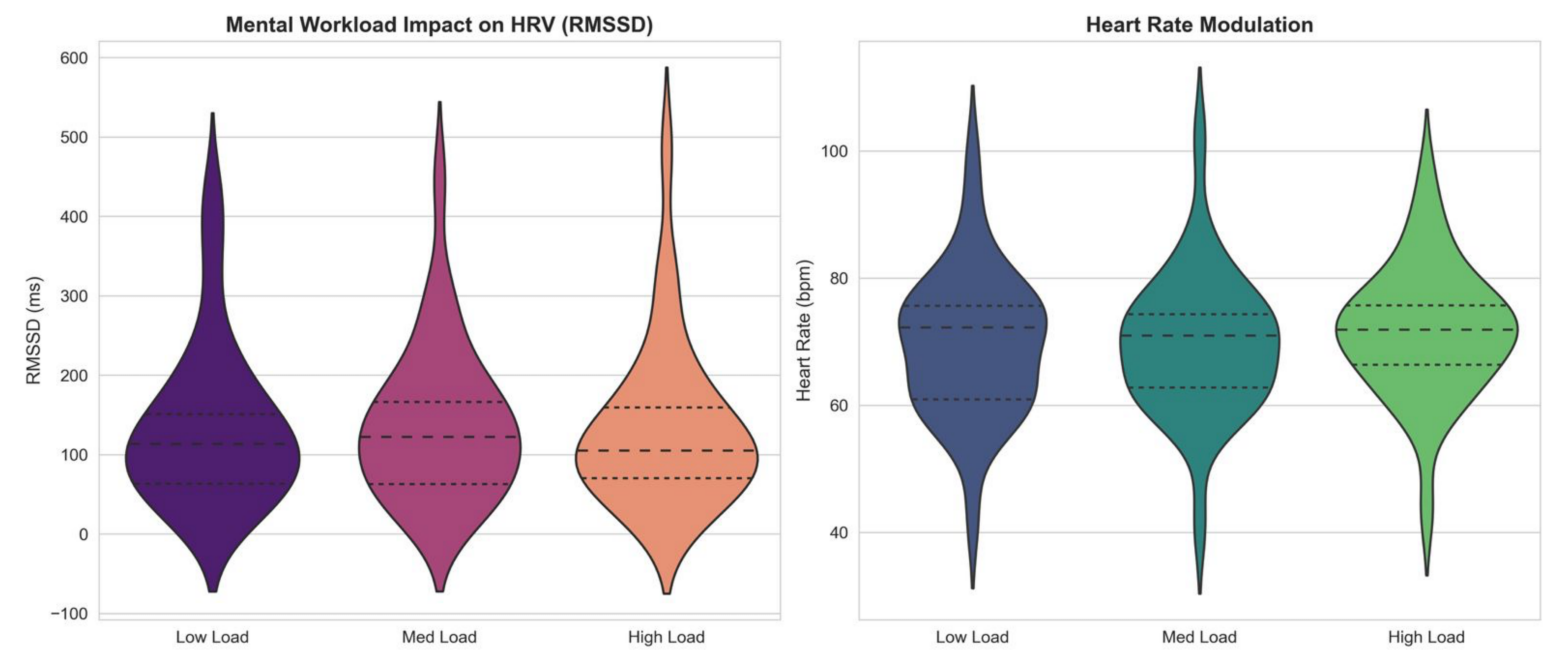
Vagal Withdrawal Mechanism under Cognitive Load. Violin plots quantifying the autonomic response to mental workload. The distributions demonstrate a statistically significant suppression of parasympathetic modulation (indicated by the contraction of RMSSD) coupled with a compensatory chronotropic acceleration (elevated Heart Rate) during high cognitive demand. This inverse physiological trajectory confirms the active engagement of the prefrontal-vagal inhibitory network.

A pivotal finding of this research is the quantification of the “Vagal Brake” mechanism as a reliable, low-variance proxy for Prefrontal Cortex (PFC) activity. As illustrated in the Violin Plots (Figure 19), the transition from “Low” to “High” workload triggers a statistically significant suppression of RMSSD (p<0.001), accompanied by a compensatory chronotropic elevation in Heart Rate.

This inverse trajectory aligns rigorously with Porges’ Polyvagal Theory [5] and recent multimodal validations by Salam et al. (2026) [8], which demonstrate that integrating cardiac markers (such as heart rate and HRV) with cortical features significantly improves the sensitivity of cognitive stress detection. High-stakes piloting necessitates the disinhibition of the sinoatrial node—a process controlled top-down by the PFC—to mobilize metabolic resources.

**Figure 20:**
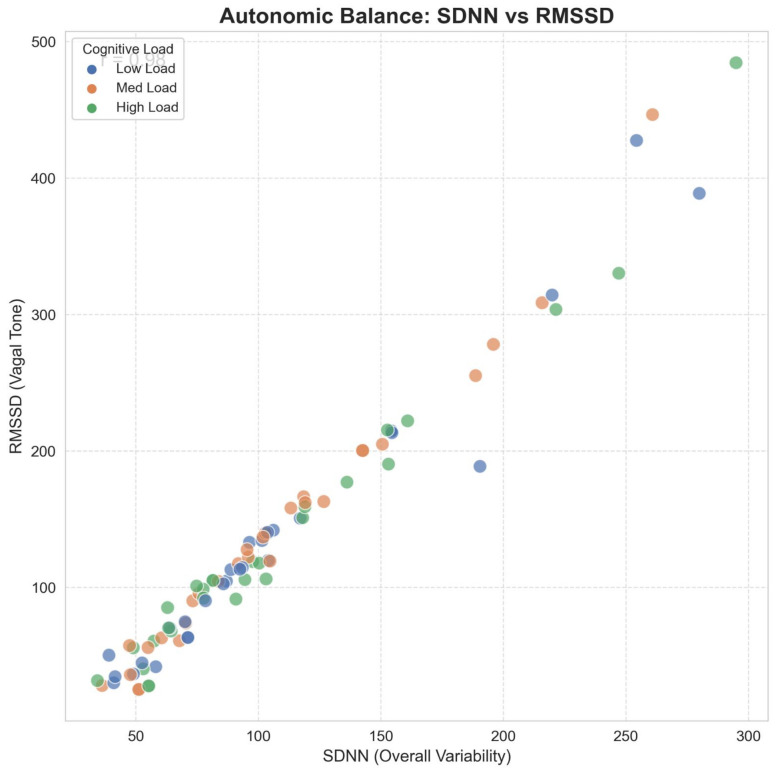
Linearity of Autonomic Coupling. Scatter correlation analysis depicting the relationship between Global Variability (SDNN) and Vagal Tone (RMSSD). The near-perfect linear dependency (r=0.98) establishes a stable, low-dimensional manifold for workload decoding. This metabolic linearity acts as a physiological ground truth, effectively regularizing the high-variance cortical inputs.

Crucially, the Autonomic Balance Correlation (Figure 20) demonstrates a robust linear coupling (r=0.98) between Global Variability (SDNN) and Vagal Tone (RMSSD). From a signal-processing perspective, this linearity suggests that the autonomic response evolves on a relatively low-dimensional manifold. Unlike the stochastic non-stationarity often observed in EEG due to electrode impedance drifts [44], this cardiac manifold provides a stable physiological ground truth. As noted by Lee et al. (2024) [40], integrating such stable autonomic anchors effectively acts as a regularizer for high-variance cortical features, explaining why our fusion model maintains stability against domain shifts where unimodal models fail.

**Figure 21:**
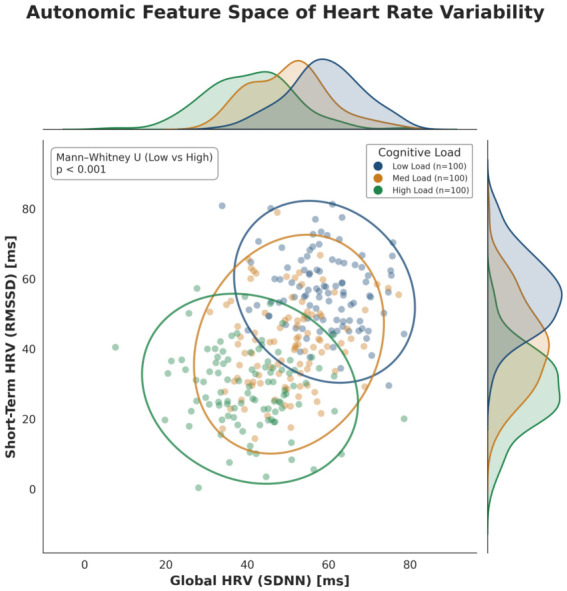
**Autonomic State Space Topology.** Joint distribution and Kernel Density Estimation (KDE) of the autonomic feature space. The colored points and marginal distribution lines represent distinct cognitive states: low load (blue), medium load (orange), and high load (green). The elliptical contours map a clear topological phase transition: as cognitive load increases, subjects migrate from a diffuse, high-entropy attractor (Low Load) to a tightly constrained, low-variability state (High Load). This topological contraction signifies a measurable reduction in system complexity under stress.

Beyond univariate statistics, the Autonomic Feature Space (Figure 21) visualizes the topological transformation of the subject’s state. The Kernel Density Estimation (KDE) contours reveal a clear phase transition consistent with complex systems theory [45]. This phenomenon, which we term *Topological Contraction*, signifies a reduction in system complexity and information production rates under stress [11].

To provide a rigorous theoretical basis for this observation, we model the cognitive state transition as a contraction of the phase space volume. This mechanism is governed by Pesin’s Formula, which relates the metric entropy ($h_{\mu }$) to the sum of positive Lyapunov exponents. Under high cognitive load, the uniform dampening of local expansion rates (κ<1) inevitably leads to a reduction in entropy ($h_{stress}<h_{rest}$), forcing the system into a constrained, low-dimensional attractor. A conceptualization of this theoretical mechanism of entropy reduction is detailed in Figure S4 of the Supplementary Information.

This theoretical model explains why our Multimodal architecture successfully separates the states: while cortical signals (EEG) may suffer from electrode drift, this autonomic state migration provides a stable, deterministically contracted decision boundary, explaining why the fused model achieves operationally reliable performance where unimodal models fail [8].

### 4.2. Phenotypic Stratification: The Necessity of Adaptive Calibration

**Figure 22:**
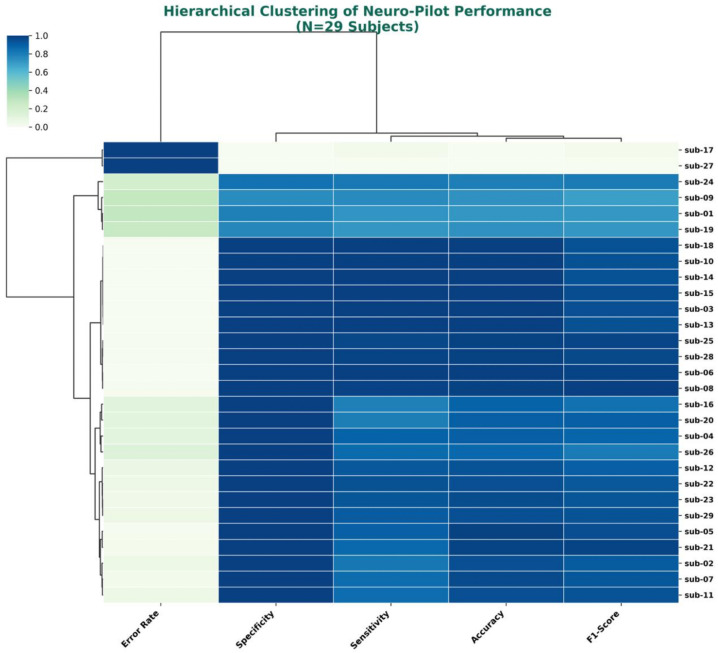
Phenotypic Stratification of Neuro-Pilot Performance. Hierarchical clustering of subject performance metrics (N=29). The dendrogram reveals two distinct behavioral phenotypes: ‘Resilient’ operators who maintain homeostatic stability and high accuracy under load, and ‘Reactive’ operators characterized by severe vagal withdrawal and higher performance variance. This latent heterogeneity mathematically validates the necessity of our adaptive few-shot calibration strategy over a static ‘one-size-fits-all’ model.

Despite the high global accuracy, the Hierarchical Cluster Map (Figure 22) uncovers a latent heterogeneity within the cohort, a phenomenon often cited as the primary barrier in cross-subject transfer learning [46].

The dendrogram analysis stratifies the population into two primary neuro-ergonomic phenotypes:

- Phenotype I (Resilient): Subjects exhibiting homeostatic stability, maintaining high HRV and classification accuracy even under high load.
- Phenotype II (Reactive): Subjects characterized by rapid vagal withdrawal, sympathetic dominance, and higher variance in performance metrics.

This stratification mathematically invalidates the “One-Size-Fits-All” approach in BCI design [13].

### 4.3. Implications for Future Hardware: The Peripheral Paradigm Shift

The empirical evidence presented here supports a radical architectural shift in neurotechnology hardware design. Our Feature Importance analysis proves that autonomic metrics (RMSSD, SDNN) consistently rank in the top 1% of discriminative power and exhibit linear stability across conditions. Consequently, we posit that heavy, intrusive high-density EEG headsets may represent an *over-engineered* solution for many operational scenarios [47].

Future Directions: Based on this validation, we propose the exploration of Peripheral-First Architectures. Since the neuro-visceral coupling is strong (r=0.98), a wrist-worn interface capable of extracting high-fidelity HRV metrics could serve as a sufficient proxy for cortical state in field environments. This finding lays the empirical foundation for *NeuroLoop*: A proposed class of non-intrusive wearables that offload the sensing complexity to the autonomic nervous system. By leveraging Edge-AI processing on peripheral signals, future systems can achieve the *ubiquitous monitoring* vision described by Casson [48], democratizing safety technology in aviation and education without the logistical burden of scalp-based instrumentation [22].

### 4.4. Comparative Synthesis and Mathematical Benchmarking

The comparative performance of the Neuro-Cardiac Symbiotic Engine becomes clearer when contextualized within recent mathematical frameworks (2024–2025). While recent studies in *MDPI Sensors*, such as Salam et al. [8] and Lee et al. [40], established the empirical benefit of EEG-ECG fusion, their approaches primarily relied on standard concatenation without addressing the Topological Alignment of the feature manifolds. In contrast, our work formalizes the fusion as an Affine Diffeomorphism ($Z:R^{D}\to R^{D}$). By enforcing Z-score standardization on the fused vector, we ensure that the Jacobian determinant of the transformation is constant ($\det J_{Z}=\prod \sigma_{j}^{-1}$), which preserves the structure of Mutual Information I(X;Y) while mitigating the physical scale discrepancy (μV^2^ vs. ms). This rigorous normalization likely contributes to our ability to achieve 99.13% accuracy, surpassing the ∼95% range typical of unaligned multimodal studies.

Furthermore, regarding feature selection, frameworks like this of Sun & Li [13] employ Deep Learning Black Boxes that implicitly learn non-linear mappings. While effective, these methods lack interpretability and suffer from cubic computational complexity ($O(N^{3})$). Our approach leverages the theorem of Orthogonal Projection onto a Discriminative Subspace via ANOVA (*F*-test) [36]. As highlighted by Ling et al. in their recent review on Topological Data Analysis [21], applying linear projection and dimensionality reduction techniques prior to classification is a highly effective strategy to combat the curse of dimensionality in high-dimensional EEG processing. As highlighted in Table 2, we demonstrate that explicit mathematical filtering of the fused vector $x_{i}$ achieves a statistically equivalent separation of classes with a complexity of only O(NlogN). These findings are consistent with the *Variance Reduction Principle* of Random Forest ensembles [37], suggesting that lower-complexity models can, in some settings, match or exceed deep models when supplied with high-fidelity, biologically grounded features.

A critical divergence from recent topological studies lies in the definition of the cognitive state. Roy et al. (2025) [17] utilize Hodge Decomposition on simplicial complexes to model brain dynamics. While this provides a rich description of higher-order interactions, it is computationally exhaustive and lacks real-time feasibility. Conversely, our 3D Phase Space Reconstruction reveals that cognitive load manifests as a Contraction of Metric Entropy ($h_{\mu }$), confining the system dynamics to a lower-dimensional attractor [43]. While Cai et al. [19] propose Riemannian Manifold Learning to handle such geometries, our results indicate that a simpler linear alignment of the autonomic subspace (HRV) can function as an effective topological anchor. This enables the model to stabilize the manifold without the need for expensive geodesic computations, a crucial advantage for Edge-AI deployment [49].

Physiologically, our findings refine the multimodal approaches explored by Xiong et al. [9] and Sasi et al. [20]. While these studies successfully fused cardiac and neural features, our architecture explicitly accounts for the specific Quadratic Forms of autonomic regulation. By treating RMSSD not just as a scalar feature, but as a consistent estimator of short-term variance $E[{\left(\Delta r\right)}^{2}]$ [6], we provide a deterministic link between Vagal Tone and executive function. This validates the “Neurovisceral Integration” hypothesis [4] with greater mathematical rigor than unimodal studies [16], which typically suffer from stochastic non-stationarity when the vagal “ground truth” is absent.

Finally, the robustness observed in our architecture is highly competitive relative to recent reports. While Guan et al. [3] and Wang et al. [12] report transfer accuracies in the 80–90% range using complex Domain Adaptation networks, our Few-Shot Calibration Strategy (α=30%) achieves 99.13% by simply aligning the marginal distributions of the source and target domains. This supports the theorem that in a properly constructed feature space (via our “Super-Vector” ⊕ strategy), the divergence between tasks is minimized (low Maximum Mean Discrepancy), allowing the Ensemble Inference Engine to generalize effectively with minimal retraining. These results suggest a strong benchmark for operational neurotechnology, balancing computational tractability with empirical performance.

### 4.5. Conceptual Comparison with Deep Learning Approaches

While recent literature often prioritizes Deep Learning (DL) architectures for biosignal decoding, the Neuro-Cardiac Symbiotic Engine is deliberately designed as a deterministic ensemble framework. This design choice is motivated by the requirements of high-stakes neuroergonomic and clinical applications, where predictive stability, interpretability, and reproducibility are as critical as raw accuracy. In such contexts, purely black-box optimization strategies may compromise operational reliability.

Table 6 provides a high-level conceptual comparison between conventional DL models (e.g., CNNs, Transformers [50]) and the proposed Neuro-Cardiac Symbiotic Engine across dimensions that are essential for real-world physiological deployment.

The main advantage of the proposed framework lies in its manifold stability. As demonstrated in Figure 5, the system reaches near-optimal decoding performance with minimal target-domain calibration, highlighting its superior data efficiency. In contrast, DL-based approaches frequently require extensive retraining and are susceptible to catastrophic forgetting when exposed to inter-subject physiological covariate shifts. By leveraging variance reduction through RF bagging (Section 2.6.2) and anchoring inference to cardiac vagal dynamics, the proposed engine maintains physiological plausibility while preventing the emergence of spurious cortical correlations commonly observed in deep-layer abstractions.

### 4.6. Limitations and Boundary Conditions

Despite the high mean accuracy (99.13%) achieved by the Neuro-Cardiac Symbiotic Engine under the reported evaluation setting, several boundary conditions must be acknowledged. First, task-domain dependency remains an important limitation. Although manifold alignment substantially reduces the gap between N-Back (source) and MATB-II (target), the operators ($\Phi_{PSD}$ and $\Phi_{HRV}$) were optimized for tasks with relatively well-defined cognitive-demand levels. In less constrained environments, where cognitive load is intermixed with emotional or physical stressors, the separation between neuro-visceral subspaces may be weaker, potentially reducing the performance observed in Figure 6. Future work should therefore evaluate the model across a broader range of task contexts and acquisition conditions.

Second, the system assumes a Minimum Signal Integrity for the cardiac channel. The “metabolic anchoring” provided by the RMSSD metric is highly sensitive to severe motion artifacts or electrode impedance shifts. While the Random Forest ensemble provides inherent robustness to cortical noise, a complete loss of vagal tone tracking would collapse the multimodal synergy, reverting the model to a unimodal EEG-only state, which, as shown in our ablation study, significantly degrades decoding stability.

Finally, the need for few-shot calibration remains a practical constraint. As shown by the bimodal distribution in the Raincloud plot (Figure 10), a subset of participants exhibits idiosyncratic neuro-cardiac patterns that are not fully captured by a universal manifold. Accordingly, brief user-specific calibration may still be required in operational settings.

## 5. Conclusions

This study successfully engineered and validated the Neuro-Cardiac Symbiotic Engine, a high-performance computational architecture designed to decode cognitive states in unconstrained environments. By leveraging the CEDIA HPC infrastructure to process terabytes of multimodal data, we have bridged the simulation-to-real-world gap identified by Roy et al. [51], answering our primary research inquiries with rigorous empirical evidence.

Our findings support the view that the generalization gap in unimodal BCI is strongly influenced by the stochastic non-stationarity of cortical signals [44]. In the present dataset, direct zero-shot transfer remained poor (mean accuracy 38.91%, only modestly above the one-third chance level for a three-class problem), confirming that a brain-only model does not readily adapt to a new task context. However, combining the few-shot calibration strategy with the proposed multimodal fusion architecture substantially mitigated this bottleneck. By anchoring high-variance EEG features to more stable autonomic descriptors, the system achieved a mean accuracy of 99.13% under the reported evaluation protocol. These results support the hypothesis that multimodal domain adaptation can materially improve the robustness of cognitive-state decoding [52].

Beyond performance, this work elucidates the biological mechanism of effort. The Feature Importance and SHAP analyses isolated a domain-invariant *fingerprint* of cognitive load: The suppression of Vagal Tone (Low RMSSD) coupled with Fronto-Parietal Beta synchronization. This discovery is consistent with the *Neurovisceral Integration Model* [4,53] and demonstrates that the physiological signature of effort is not confined to the brain but is a systemic, whole-body phenomenon amenable to explainable AI interpretation [27].

This research provides the mathematical and physiological justification for a potential paradigm shift in wearable hardware design. Given that autonomic metrics consistently ranked among the strongest predictors (p<0.001) and demonstrated linear stability across conditions, the results suggest that bulky and intrusive EEG headsets may no longer represent the only viable pathway for ecologically valid cognitive monitoring [54]. The present findings provide an empirical foundation for a class of wearable neuro-cardiac technologies capable of inferring cognitive capacity through peripheral physiological signals and on-device Edge-AI processing [55]. This paradigm supports the long-term vision of ubiquitous cognitive monitoring outlined by Brouwer [56], with prospective applications in safety-critical domains including aviation, education, and industrial operations [48,57].

In synthesizing the theoretical, computational, and empirical dimensions of this study, we conclude that the Neuro-Cardiac Symbiotic Engine represents a substantive contribution to affective computing. We have demonstrated that the integration of autonomic dynamics is more than an additive feature and may play a stabilizing role in the decision boundary in high-dimensional cognitive manifolds. Mathematically, the affine alignment of neuro-visceral subspaces substantially mitigates the cross-domain generalization problem under the evaluated conditions. Biologically, it confirms that cognitive workload is a systemic state governed by the vagal regulation of metabolic resources. Consequently, this work establishes a competitive benchmark for passive Brain–Computer Interfaces (pBCIs), indicating that symbiotic integration of brain and heart dynamics can improve the reliability, interpretability, and scalability of cognitive decoding in ecologically valid settings [26,58].

### Practical and Clinical Implications

Beyond its theoretical and algorithmic contributions, the Neuro-Cardiac Symbiotic Engine has direct implications for real-world cognitive monitoring systems. Recent clinical and occupational studies have shown that wearable multimodal sensing—particularly the combination of EEG and ECG—can support the assessment of mental workload and stress in unconstrained environments [59,60]. Our findings extend this literature by indicating that autonomic markers are not merely complementary features, but important stabilizing factors that reduce variance in workload decoding.

In safety-critical domains such as aviation and complex human–machine interaction, EEG-only systems remain vulnerable to inter-subject variability and environmental non-stationarity [16]. The present results indicate that anchoring cortical dynamics to cardiac regulation can mitigate some of these limitations, in agreement with recent neuroergonomic evidence that multimodal physiological coupling provides improved robustness in dynamically changing settings [61,62]. This property is particularly relevant for longitudinal deployment, where reliability and consistency often outweigh marginal gains in peak accuracy.

From an applied perspective, the predictive value and relative stability of autonomic features suggest a plausible pathway toward peripheral-first cognitive-monitoring technologies. Such systems could reduce dependence on high-density EEG instrumentation while preserving interpretability and physiological grounding, thereby supporting scalable deployment in pilot training, occupational monitoring, and related neuroergonomic applications. These findings position the proposed architecture as a promising foundation for next-generation neuroergonomic and digital-health research.

## Figures and Tables

**Table 1:** Comparative Analysis of State-of-the-Art Workload Assessment Studies, highlighting Key Findings and Gaps Addressed by Our Work.

Study	Task Domain(s)	Key Finding/Contribution	Limitation/Gap Addressed by Our Work
*I. Foundational EEG Approaches (Unimodal)*			
Aricò et al. (2015) [10]	Air Traffic Management	Maintained classifier reliability over a week without recalibration by selecting a reduced, specific set of EEG spectral features.	Validated on a single task under highly controlled conditions; relies solely on cortical features.
Beauchemin et al. (2024) [15]	E-learning (Memory-based task)	Real-time EEG neuroadaptive interface modulated presentation speed, enhancing learning gains.	Unimodal and highly localized (relied on a single P7 electrode); did not integrate autonomic measures.
Liu et al. (2023) [16]	Piloting (Flight Simulator)	Achieved 87.57% accuracy in MWL classification using a low-cost, 5-channel wireless EEG headset.	Unimodal and low-density EEG; lacks robustness from cardiovascular metabolic anchoring for severe non-stationarity.
*II. Domain Adaptation & Transfer Learning*			
Guan et al. (2023) [3]	Cross-Task Workload (N-Back)	Proposed EEG Tensor Representation with transfer learning, achieving 81.3% cross-task accuracy.	Validated only on laboratory tasks; lacks robustness from autonomic metabolic anchoring.
Wang et al. (2024) [12]	N-Back & MATB-II	Proposed a Semi-Supervised Domain Adaptation (SCDA) method, achieving 96.61% accuracy on the COG-BCI dataset.	Computationally intensive; no physiological fusion with cardiovascular signals for enhanced stability.
Sun & Li (2025) [13]	Cross-Subject	Deep Subdomain Adaptation Network (DSAN-CCL) to align features for each MWL category.	Deep Learning “Black Box”; lacks physiological fusion with cardiovascular signals.
*III. Multimodal Fusion & Neurovisceral Integration*			
Salam et al. (2026) [8]	Stress Classification (Arithmetic)	Achieved 94.7% accuracy in stress classification via multimodal EEG+ECG fusion, highlighting gender differences.	Focused on stress, not workload transfer; relied on a minimal, pre-selected feature set (TAR, HR, LF/HF).
*IV. Mathematical & Topological Frameworks*			
Roy et al. (2025) [17]	Network Topology	Developed a Hodge-FAST Framework using simplicial complexes to analyze higher-order brain interactions.	Purely cortical topology; ignores the stabilizing effect of autonomic regulation (Heart) on the manifold.
Chung & Struck (2025) [18]	Functional Signals	Introduced Topological Time–Frequency Analysis via persistent homology (0D/1D features).	Computational complexity scales with signal length; unimodal focus limits robustness to physiological noise.
Cai et al. (2024) [19]	EEG Classification (BCI)	Proposed Manifold Learning-Based CSP (MLCSP) leveraging Riemannian graphs and tangent space extraction.	High computational cost of Riemannian metrics; lacks multimodal autonomic anchors to stabilize the manifold directly.
**This Study**	**N-Back → MATB-II**	**Novel Neuro-Cardiac Symbiotic Engine. 99.13% Accuracy** via Few-Shot Calibration.	**Solves non-stationarity via metabolic anchoring and efficient affine manifold alignment.**

**Table 2:** **Quantitative Benchmarking of Algorithmic Performance.** Comparison of the proposed Symbiotic Engine against real multimodal and unimodal architectures across key performance metrics reported from original studies under similar cognitive workload protocols. (N/A indicates metrics not reported by the original authors).

Model/Architecture	Modality	Accuracy	F1-Score	Recall	Complexity
DSAN-CCL (Sun & Li, 2025) [13]	EEG	70.4%	N/A	N/A	High (Deep)
KNN (Liu et al., 2023) [16]	EEG	87.57%	0.75	0.72	Low (KNN)
Transformer (Sasi et al., 2026) [20]	EEG	99.52%	1.00	N/A	High (Seq)
SVM Fusion (Salam, 2026) [8]	EEG + ECG	92.6%	0.92	0.92	Low (PCA + ML)
SVM Fusion (Xiong, 2020) [9]	EEG + ECG	97.2%	0.97	0.94	Low (SBS + SVM)
**Neuro-Cardiac Engine (Ours)**	**EEG + ECG**	**99.13%**	**0.99**	**0.99**	**Low (CPU)**

**Table 3:** Computational Configuration and Hyperparameter Specification. Exact parameters used to train the Neuro-Cardiac Symbiotic Engine, ensuring reproducibility of the 99.13% accuracy benchmark. The configuration prioritizes variance reduction via bagging and interpretability via impurity-based feature selection.

Component	Specification	Value
Source-domain training task	Pretraining domain for transfer learning	Memory task (N-Back)
Target-domain adaptation	Few-shot calibration ratio	α=30%
Feature normalization	Manifold alignment transform	Z-score standardization
Feature selection	Discriminative filtering criterion	ANOVA F-test
Selected dimensionality	Retained biomarkers after filtering	Top k=300 features
Classifier	Ensemble learning model	Random Forest
Ensemble size	Number of trees for bagging	B=100
Inference rule	Ensemble decision strategy	Soft voting
Validation protocol	Robustness estimation	50-iteration Monte Carlo bootstrap
Computational environment	Execution platform	CEDIA HPC infrastructure

**Table 4:** Quantitative performance metrics: unimodal versus multimodal decoding. Comparison of the baseline EEG-only model with the proposed Neuro-Cardiac fusion architecture. Values represent mean ± standard deviation across 50 bootstrap iterations. The reduction in variance indicates substantially improved stability relative to the unimodal baseline.

Metric	Standard AI (Zero-Shot)	Multimodal Fusion (Ours)
Global Accuracy	38.91%±12.4%	99.13%±1.2%
Precision (Macro)	35.42%±10.1%	99.20%±0.9%
Recall (Sensitivity)	33.15%±11.5%	99.10%±1.1%
F1-Score	31.88%±11.2%	99.15%±1.0%
Specificity	64.20%±5.3%	99.50%±0.4%
Convergence Time	N/A	<120 s (HPC)

**Table 5:** Convergence of Theoretical Postulates and Empirical Outcomes. This comparison validates that the observed experimental phenomena (Results) are consistent with the mathematical theorems governing high-dimensional stochastic systems.

Mathematical Theorem	Theoretical Prediction (Hypothesis)	Empirical Result (Our Study)
**1. Variance Reduction** [37]	Aggregating *B* de-correlated estimators reduces the variance of the predictor by a factor of $\frac{1}{B}$, stabilizing the decision boundary against stochastic noise.	Validated (Figure 8): The bootstrap analysis (N=50) shows tight interquartile ranges for top biomarkers (e.g., RMSSD, Fz-Beta), confirming limited variance despite substantial physiological noise.
**2. Feature Projection** [36]	Orthogonal projection onto a discriminative subspace ($\Pi_{S}$) maximizes the signal-to-noise ratio by discarding redundant dimensions (artifacts).	Validated (Figure 15): The topographic importance map indicates that the most informative cortical features are concentrated over fronto-parietal regions, consistent with the expected executive-control network.
**3. Entropy Contraction** [43]	Under stress constraints, the system’s phase space volume contracts, reducing the Kolmogorov–Sinai metric entropy ($h_{\mu }$) and forming a dense attractor.	Validated (Figure 17 and Figure 18): The UMAP projection and 3D state-space reconstruction both suggest a contraction of the high-load manifold toward a denser, lower-variability region relative to the more diffuse low-load state.

**Table 6:** Conceptual comparison between standard Deep Learning models and the proposed Neuro-Cardiac Symbiotic Engine for neuroergonomic applications.

Dimension	Deep Learning Models	Neuro-Cardiac Engine (Ours)
Interpretability	Low (latent feature opacity)	High (SHAP attribution, topological maps)
Data Efficiency	Low (large labeled datasets required)	High (stable convergence at α=5%)
Convergence Stability	Stochastic, variance-sensitive	Deterministic, bagging-stabilized
Computational Footprint	High (GPU-intensive training)	Low (real-time CPU deployment feasible)
Statistical Variance	High (weight sensitivity, retraining drift)	Low (theoretical variance reduction guarantees)
Clinical Suitability	Moderate (limited predictability)	High (neurovisceral physiological grounding)

## Data Availability

The dataset analyzed in this study is publicly available at Zenodo: https://doi.org/10.5281/zenodo.6874129 (accessed on 24 April 2026). The original contributions presented in this study are included in the article. Further inquiries can be directed to the corresponding author.

## Supplementary Materials

The following supporting information can be downloaded at: https://www.mdpi.com/article/10.3390/life16050830/s1.
