# Supplementary material for: The Neuro-Cardiac Symbiotic Engine: A Multimodal Fusion Architecture for Cognitive State Decoding via High-Performance Computing
Source: Life (Basel). 2026 May 18;16(5):830. doi: 10.3390/life16050830 (PMC13208311; doi:10.3390/life16050830)
Supplement: Supplementary file 1 [file life-16-00830-s001.zip › life-4246624-Supplementary.pdf]

# Supplementary Information

## The Neuro-Cardiac Symbiotic Engine: A Multimodal Fusion Architecture for Cognitive State Decoding via High-Performance Computing

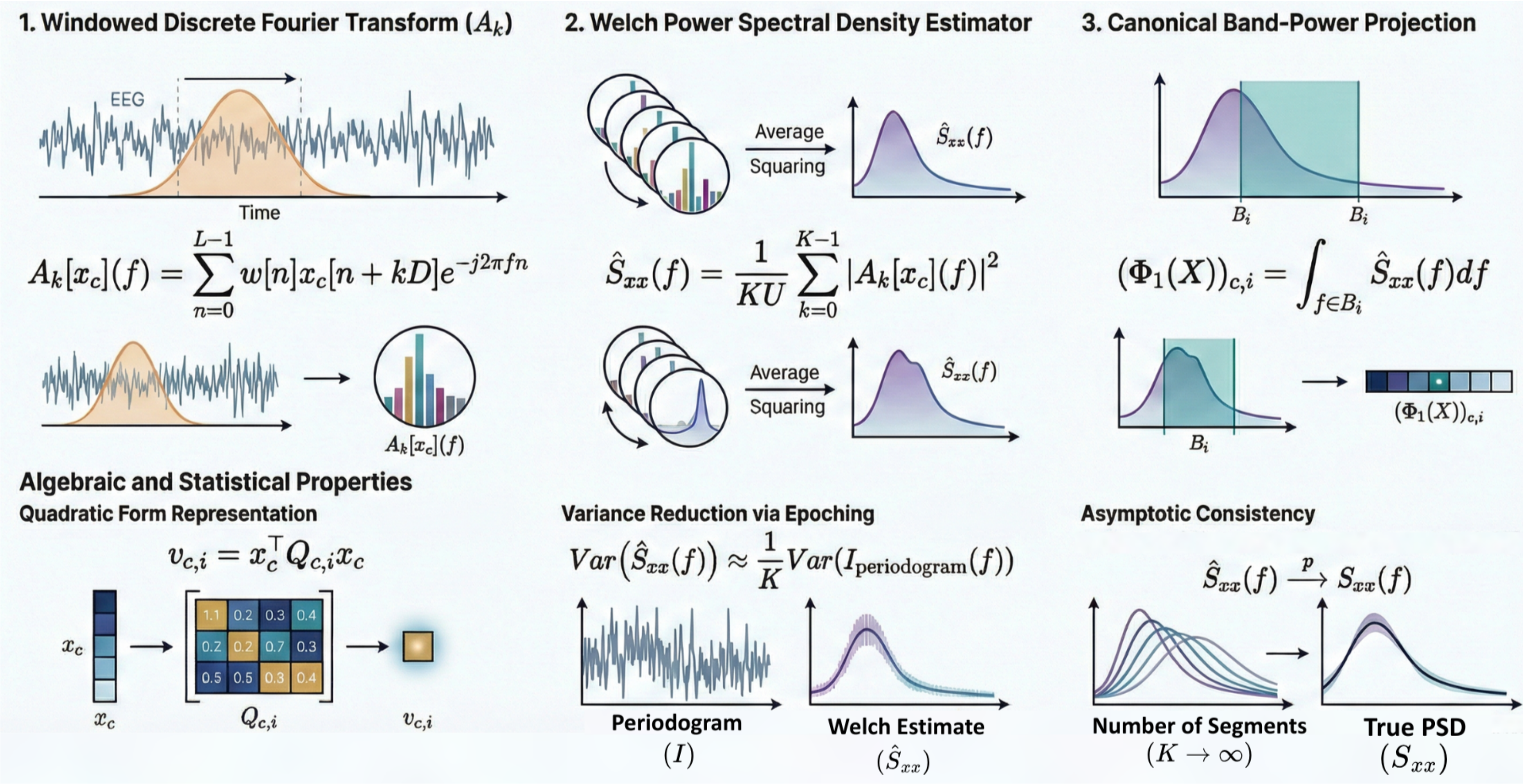


Figure S1: **The Mathematical Architecture of the Phi1 Spectral Operator.** Visual decomposition of the cortical feature extraction pipeline. **(Top Panels)** The raw signal undergoes a windowed discrete Fourier transform, followed by squared-magnitude averaging to form the Welch periodogram estimate. **(Bottom Left)** Algebraic formulation demonstrating that band-power extraction is a positive semi-definite quadratic form. **(Bottom Center)** Variance reduction principle: averaging segments reduces stochastic noise proportionally. **(Bottom Right)** Asymptotic consistency showing convergence to the true spectrum as segment count increases.

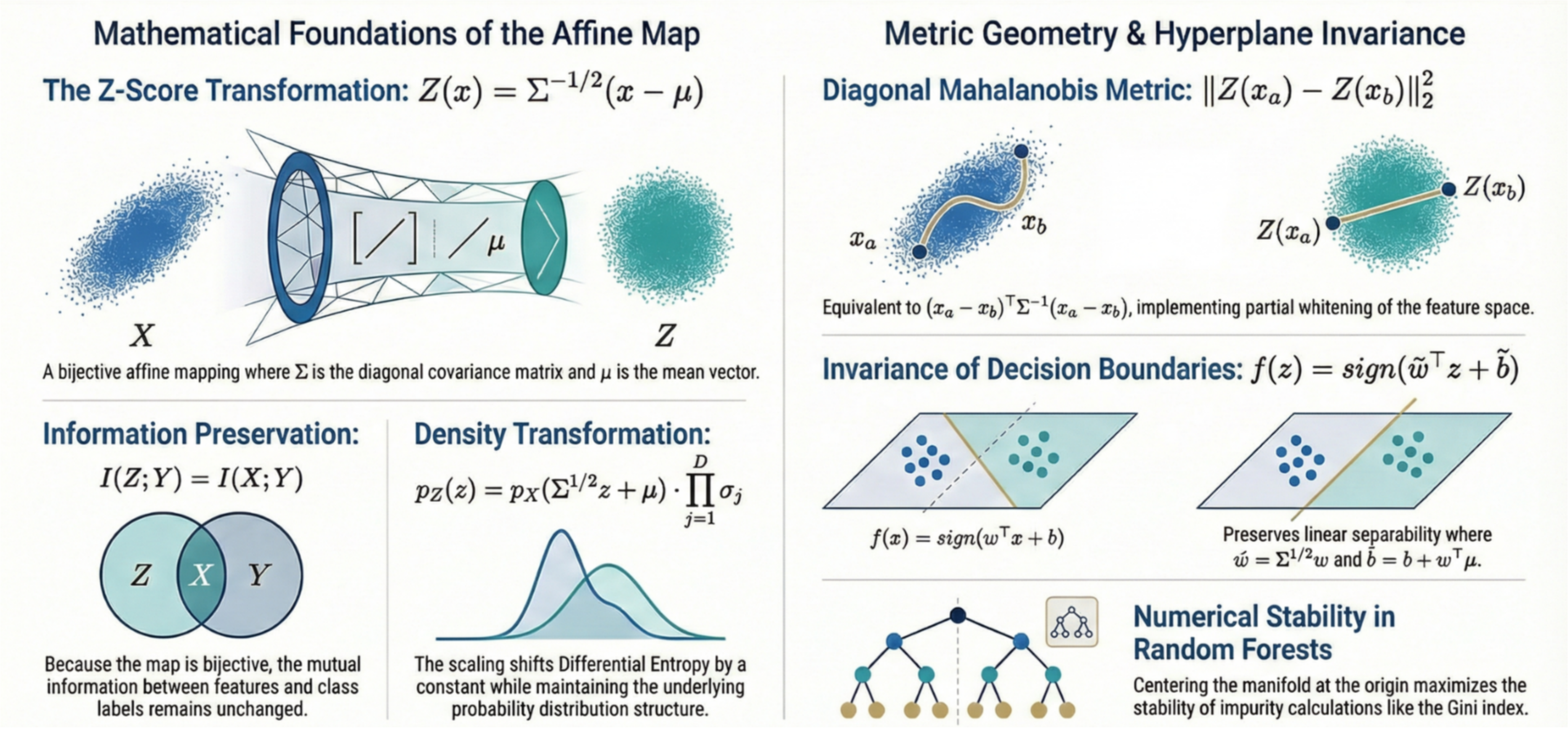


Figure S2: **The Geometry of Affine Feature Normalization.** Visual formalization of the Z-score transformation as a topology-preserving map. **(Left)** The transformation is depicted as an affine diffeomorphism that reshapes the data distribution without losing information. **(Right)** Geometric implications: Euclidean distances in the normalized space (Z) correspond to diagonal Mahalanobis distances in the original space (X), effectively implementing a partial whitening. This maximizes the numerical stability of the Random Forest split criteria.

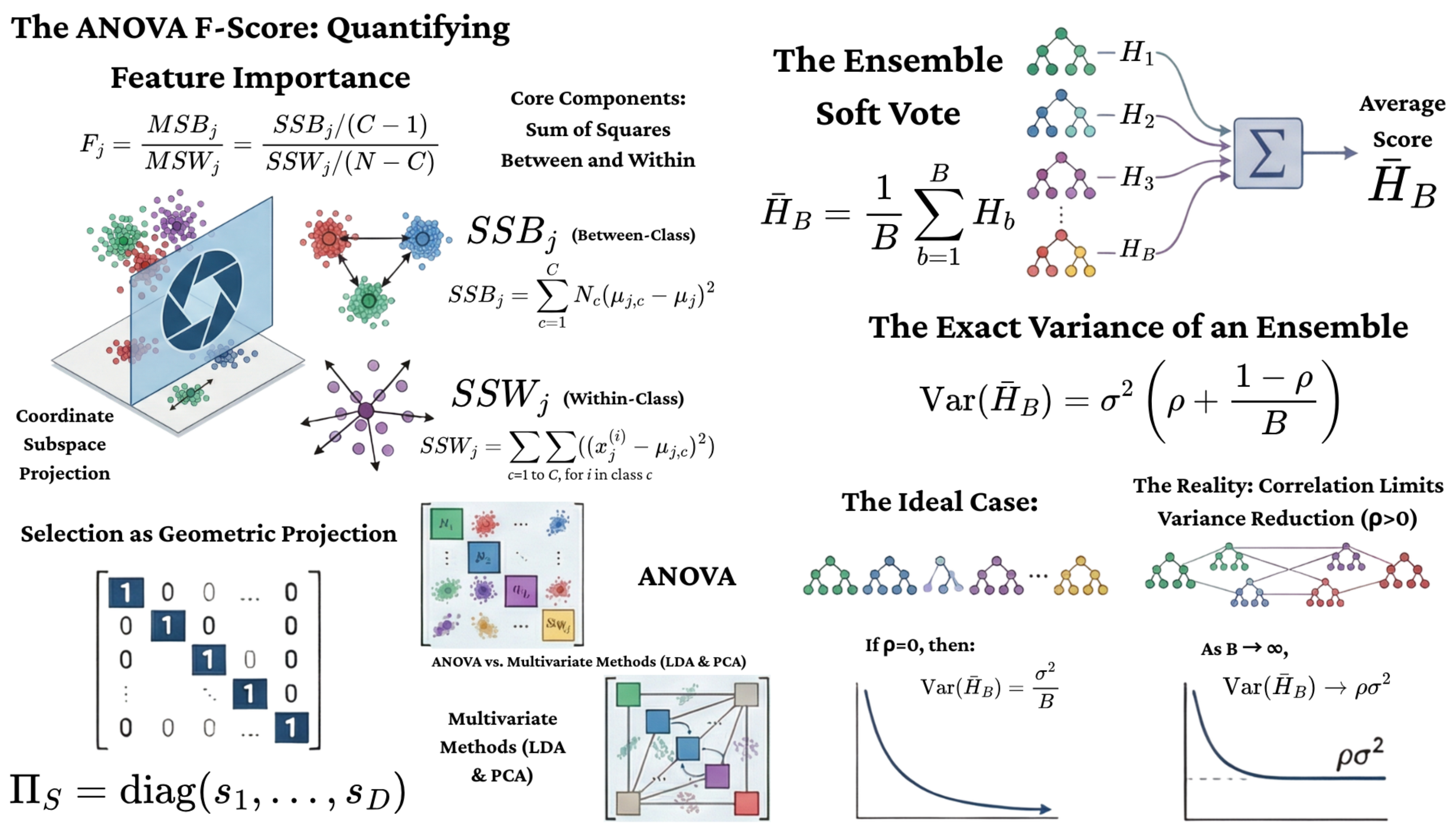


Figure S3: **Mathematical architecture of the learning engine: from projection to inference. (Left panel)** Recapitulation of the ANOVA-based projection logic. The F-statistic ($F_j$) is derived from the ratio of between-class ($SSB_j$) and within-class ($SSW_j$) sum of squares, formulating the diagonal projection matrix $\Pi_S$. **(Right panel)** Variance-reduction principle of the Random Forest ensemble. The soft-voting consensus acts as a stable estimator. As the number of trees $B \to \infty$, the ensemble variance decays asymptotically toward the irreducible limit imposed by the pairwise tree correlation ($\rho\sigma^2$).

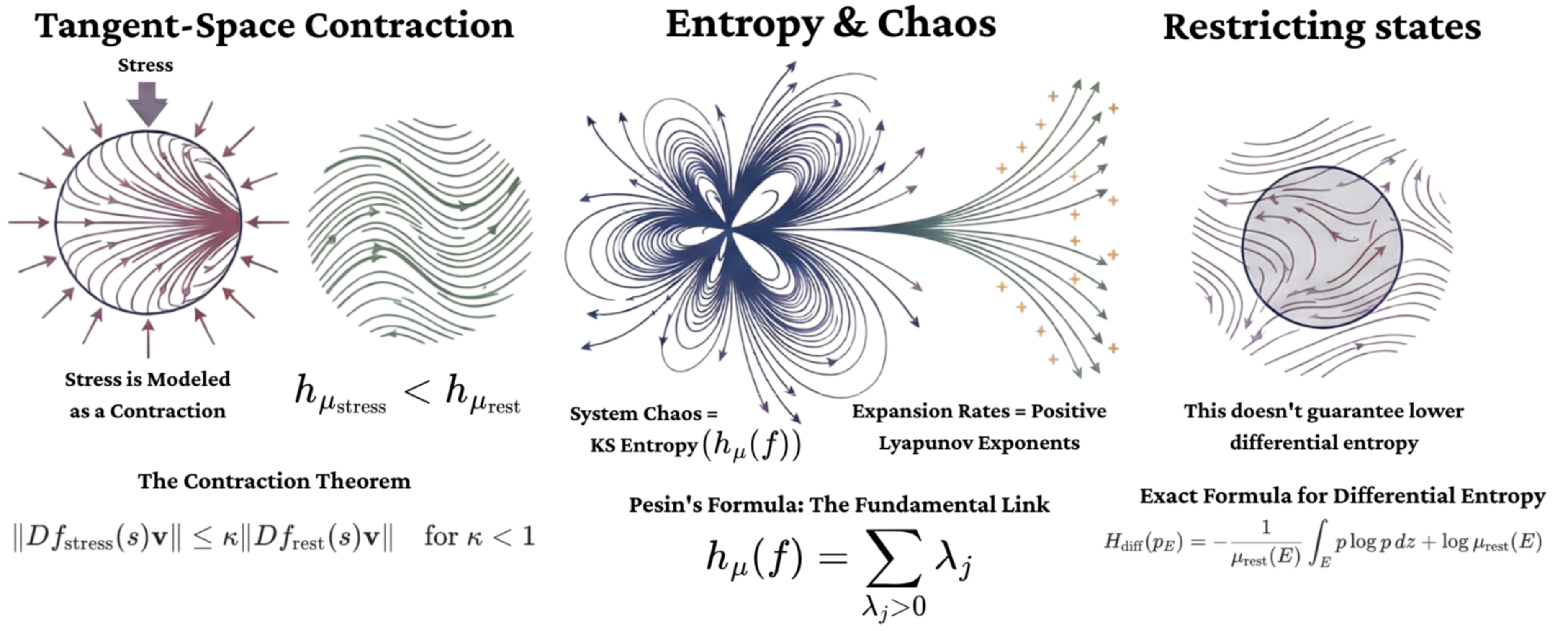


Figure S4: **Theoretical mechanisms of entropy reduction. (Left panel: Entropy & Chaos)** Conceptualization of Kolmogorov-Sinai (KS) metric entropy ($h_\mu(f)$) as a measure of the average rate of information production in a dynamical system. Pesin's formula provides the fundamental link, rigorously equating entropy to the sum of positive Lyapunov exponents. **(Center panel: Tangent-Space Contraction)** Stress is modeled as a uniform contraction. A stress dynamic is assumed to uniformly dampen the system's local expansion rates. **(Right panel: Restricting States)** Measure restriction as a contrasting mechanism. Restricting the state space to a subset $E$ modifies differential entropy.
